# Disrupting the α-synuclein-ESCRT interaction with a peptide inhibitor mitigates neurodegeneration in preclinical models of Parkinson’s disease

**DOI:** 10.1038/s41467-023-37464-2

**Published:** 2023-04-19

**Authors:** Satra Nim, Darren M. O’Hara, Carles Corbi-Verge, Albert Perez-Riba, Kazuko Fujisawa, Minesh Kapadia, Hien Chau, Federica Albanese, Grishma Pawar, Mitchell L. De Snoo, Sophie G. Ngana, Jisun Kim, Omar M. A. El-Agnaf, Enrico Rennella, Lewis E. Kay, Suneil K. Kalia, Lorraine V. Kalia, Philip M. Kim

**Affiliations:** 1 Donnelly Centre for Cellular and Biomolecular Research, University of Toronto, Toronto, ON Canada; 2 Krembil Research Institute, Toronto Western Hospital, University Health Network, Toronto, ON Canada; 3 Neurological Disorders Research Center, Qatar Biomedical Research Institute (QBRI), Hamad Bin Khalifa University (HBKU), Qatar Foundation, Doha, Qatar; 4 Department of Molecular Genetics, University of Toronto, Toronto, ON Canada; 5 Department of Biochemistry, University of Toronto, Toronto, ON Canada; 6 Department of Chemistry, University of Toronto, Toronto, ON Canada; 7 Program in Molecular Medicine, The Hospital for Sick Children Research Institute, Toronto, ON Canada; 8 Division of Neurosurgery, Department of Surgery, University of Toronto, Toronto, ON Canada; 9 Division of Neurology, Department of Medicine, University of Toronto, Toronto, ON Canada; 10 Tanz Centre for Research in Neurodegenerative Diseases, University of Toronto, Toronto, ON Canada; 11 Department of Computer Science, University of Toronto, Toronto, ON Canada

**Keywords:** Cell death in the nervous system, Networks and systems biology, Recombinant peptide therapy, Parkinson's disease

## Abstract

Accumulation of α-synuclein into toxic oligomers or fibrils is implicated in dopaminergic neurodegeneration in Parkinson’s disease. Here we performed a high-throughput, proteome-wide peptide screen to identify protein-protein interaction inhibitors that reduce α-synuclein oligomer levels and their associated cytotoxicity. We find that the most potent peptide inhibitor disrupts the direct interaction between the C-terminal region of α-synuclein and CHarged Multivesicular body Protein 2B (CHMP2B), a component of the Endosomal Sorting Complex Required for Transport-III (ESCRT-III). We show that α-synuclein impedes endolysosomal activity via this interaction, thereby inhibiting its own degradation. Conversely, the peptide inhibitor restores endolysosomal function and thereby decreases α-synuclein levels in multiple models, including female and male human cells harboring disease-causing α-synuclein mutations. Furthermore, the peptide inhibitor protects dopaminergic neurons from α-synuclein-mediated degeneration in hermaphroditic *C. elegans* and preclinical Parkinson’s disease models using female rats. Thus, the α-synuclein-CHMP2B interaction is a potential therapeutic target for neurodegenerative disorders.

## Abstract (web summary)

ESCRT-III is involved in the endolysosomal system and disturbed in neurodegenerative diseases. Here the authors show that disruption of an interaction between ESCRT-III member CHMP2B and α-synuclein by a peptide inhibitor mitigates neurodegeneration in Parkinson’s disease models.

## Introduction

Protein-protein interactions (PPIs) govern virtually all molecular pathways involved in cell growth, differentiation, and survival^1,2^. Inhibition of PPIs with peptides or small molecules to modulate these pathways has proven to be successful for the treatment of cancers^3,4^. PPI inhibitors could conceivably be a promising new therapeutic venue for neurodegenerative disorders, such as Parkinson’s disease (PD), for which no disease-modifying therapies exist^5^.

Wild-type (WT) or mutant α-synuclein protein (a-syn) accumulates in PD to form oligomers that disrupt core cellular systems causing neurodegeneration^6^. Rescuing these toxic effects by targeting PPIs has been an unexplored therapeutic strategy for PD^7,8^. However, development of PPI inhibitors that reduce a-syn accumulation will be excessively prolonged if we rely on current strategies based on targeting individual interactions revealed solely by serendipitous discoveries^9^.

To expedite the discovery of potential peptide therapies, we recently developed a screening system to find candidate peptides that perturb endogenous PPIs. We identified putative PPI inhibitors using computational methods based on PPI motifs and constructed lentiviral libraries of these peptide inhibitors. These libraries contain short linear interaction motifs that mediate a large fraction (~30%) of human PPIs^10–12^.

In this work, we use this unbiased and proteome-wide approach to identify PPI inhibitors that reduce a-syn-mediated neurodegeneration. We discover a peptide inhibitor that disrupts the direct interaction between the C-terminal region of a-syn and CHarged Multivesicular body Protein 2B (CHMP2B). This interaction impairs endolysosomal activity resulting in a-syn accumulation. We demonstrate that the peptide inhibitor restores endolysosomal function and thereby reduces a-syn levels, rescuing dopaminergic neurons from degeneration. Based on our findings, we conclude that the a-syn-CHMP2B interaction is a potential therapeutic target for PD and other neurodegenerative conditions.

## Results

### Proteomic screen identifies peptides that rescue a-syn cytotoxicity and reduce a-syn oligomers

**Fig. 1:**
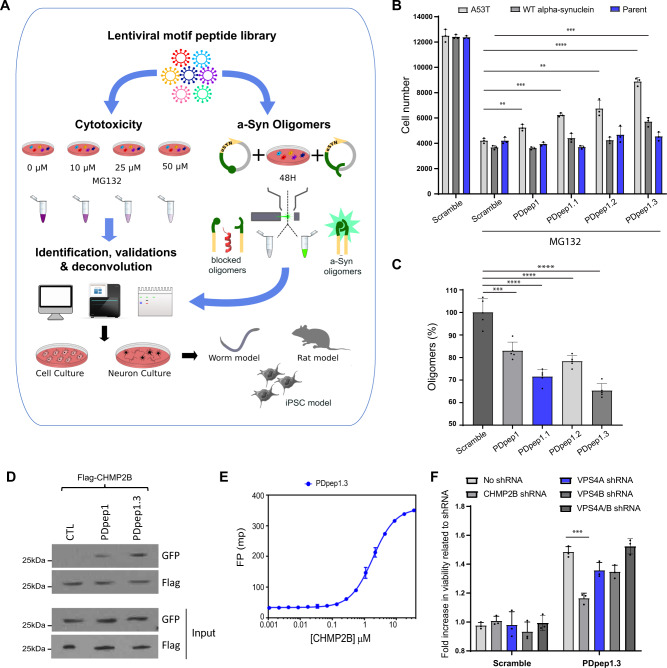
Proteomic screens and in vitro validation of hits. **A** Schematic of the experimental design including proteomic screens, identification and validation of hits, target deconvolution, and evaluation in cell and animal models (created using Servier Medical Art at https://smart.servier.com/). For the cytotoxicity screen, we employed our peptide library to identify peptides that rescue cytotoxicity induced by a-syn overexpression and proteostatic stress (due to MG132 administration). To screen our library for peptides that inhibit a-syn oligomers, we used FACS with a split YFP-a-syn system (cells co-expressing V1S and SV2). **B** Validation of cell viability effect of peptides in A53T and WT a-syn expressing cells under proteostatic stress as well as controls. MG132 was used at a concentration of 10 μM. Experiments were done in triplicate. Data represent means ± s.d. (unpaired two-tailed *t*-tests; A53T PDpep1, *P* = 0.0051; A53T PDpep1.1, *P* = 0.0002; A53T PDpep1.2, *P* = 0.0035; A53T PDpep1.3, *P* < 0.0001; WT a-syn PDpep1.3, *P* = 0.0008; *n* = 3). **C** WT a-syn oligomers as measured by luciferase activity. All four peptides showed significant reduction in a-syn oligomers from cells stably expressing split luciferase-a-syn constructs. Experiments were done in triplicate. Data represent means ± s.d. (unpaired two-tailed *t*-tests; PDpep1, *P* = 0.0008; PDpep1.1, PDpep1.2, PDpep1.3, *P* < 0.0001; *n* = 3). **D** Co-immunoprecipitation experiment with Flag-CHMP2B and GFP-peptides (*n* = 3). PDpep1 and PDpep1.3 peptides interacted with Flag-CHMP2B whereas GFP alone (CTL) did not. **E** Fluorescence polarization (FP) binding assay of a FITC-labeled PDpep1.3 peptide against CHMP2B. Error bars represent ± s.d. of the fit (*n* = 5). **F** Knockdown of CHMP2B or VPS4 in A53T a-syn expressing cells under proteostatic stress. Cell viabilities were measured with cells stably expressing different shRNAs and transfected with Scramble or PDpep1.3 peptide. Experiments were done in triplicate. Data represent means ± s.d. (unpaired two-tailed *t*-tests; PDpep1.3, *P* = 0.0005; *n* = 3). **P < 0.01; ***P < 0.001; ****P < 0.0001. Source data are provided as a Source Data file.

We used a lentiviral library of 50,549 7-mer peptides enriched in PPI motifs^13^ on a green fluorescent protein (GFP) scaffold (Fig. 1A). We infected cells in a pooled format and the amino acid sequence of each peptide served as its own barcode. By using a low multiplicity of infection followed by puromycin selection, each infected cell expressed one unique GFP-peptide. We screened for peptides that reduced a-syn-mediated cytotoxicity or a-syn oligomers in parallel (Fig. 1A). To cause cytotoxicity relevant to PD, we induced proteostatic stress in HEK293 cells by inhibiting the proteasome with MG132 and overexpressing human WT a-syn associated with idiopathic PD in one screen or the more toxic mutant A53T a-syn associated with familial PD in a second screen. This combination of a-syn overexpression and pharmacological proteasome inhibition led to death of most cells, with only those that expressed a protective peptide surviving. By extracting genomic DNA from the surviving cells and amplifying the peptide coding sequences, we identified the protective peptides (Fig. 1A). To detect a-syn oligomers, we used a protein fragment complementation assay (PCA) of a-syn oligomerization in HEK293 cells^14^. In this assay, human A53T a-syn was fused to either the C- or N-terminal half of yellow fluorescent protein (YFP). No fluorescence is detectable while a-syn exists in monomers but, when a-syn oligomerizes, the two halves of YFP come in close enough proximity to form a functional, spontaneously fluorescent protein. This fluorescence has previously been shown to approximate levels of toxic oligomeric conformations of a-syn in cell and rodent models^15–18^. By using fluorescence-activated cell sorting (FACS), we isolated cells expressing Flag-tagged peptides that directly or indirectly interfered with a-syn oligomer formation and then identified the peptides from genomic DNA (Fig. 1A). Our screens yielded 10 hits (Table S1). The hit with the most reads (i.e., the most abundant) was the same peptide in all screens: IPIQLKA (named PDpep1) (Table S1). We validated the cytoprotective effects of PDpep1 in HEK293 cells overexpressing WT or A53T a-syn (Figs. 1B, S1A), as well as its ability to reduce WT or A53T a-syn oligomers in a luciferase-based PCA^18–20^ compared to a scrambled control peptide (Figs. 1C, S1B).

### Lead peptide targets CHMP2B, a member of ESCRT-III

We mapped the amino acid sequence of PDpep1 to the C-terminal region of CHMP2B, a member of the Endosomal Sorting Complex Required for Transport-III (ESCRT-III) machinery. ESCRT is involved in the endolysosomal system and other fundamental cellular processes. The endolysosomal system is made of dynamic organelles (early endosomes, recycling endosomes, late endosomes, and lysosomes) into which cargo molecules such as proteins are internalized, transported, recycled, or digested to maintain cellular homeostasis. Autophagy converges on the later stages of the endolysosomal system; specifically, autophagosomes fuse with late endosomes or lysosomes resulting in autophagic degradation of unnecessary or dysfunctional cellular components. The peptide we mapped to CHMP2B corresponds to a MIT-Interacting Motif (MIM) (Fig. S1C)^21^ that is thought to mediate several PPIs. Using this mapping, we sought to optimize PDpep1, hypothesizing that our initial hit only weakly resembles the actual motif and only a fragment of it. To do this, we generated several versions of the peptide of various lengths based on the relevant sequence of CHMP2B: IERQLKA (PDpep1.1), EIERQLKALG (PDpep1.2), and DEEIERQLKALG (PDpep1.3) (Fig. S1C). We found that PDpep1.3 had the most prominent effects on cell survival under proteostatic stress due to MG132 treatment and overexpression of WT or A53T a-syn (Fig. 1B) and on WT or A53T a-syn oligomers (Figs. 1C, S1B), consistent with our hypothesis.

Many ESCRT members have been implicated in neurodegenerative proteinopathies, such as Hrs of ESCRT-0 and Tsg101 of ESCRT-I in models of Alzheimer’s disease^22^. One obvious mechanistic link to proteinopathies is the key role ESCRT plays in protein degradation by the endolysosomal pathway. ESCRT controls formation of multivesicular bodies (MVBs), a subset of late endosomes that contain cargo-laden intralumenal vesicles (ILVs). MVBs can fuse with lysosomes leading to degradation of ILVs and their protein cargoes. In addition, ESCRT-III is important for lysosome maintenance and repair of lysosomal membranes^23^. Mutations in the *CHMP2B* gene are rare but established causes of amyotrophic lateral sclerosis (ALS) and frontotemporal dementia (FTD)^24,25^. A genome-wide association study has also nominated a *CHMP2B* variant as a genetic risk factor for PD^26^. The CHMP2B protein is expressed in all neurons, and brains of mice expressing mutant CHMP2B form neuronal inclusions due to impaired lysosomal degradation^27^. Furthermore, previous studies found that CHMP2B co-localizes with a-syn in Lewy bodies in brains of PD patients^28^ and that a-syn co-immunoprecipitates and co-localizes with CHMP2B in transgenic a-syn mouse brains and human brains with Lewy bodies^29^.

CHMP2B has been reported to bind to Vacuolar Protein Sorting 4 (VPS4), an AAA-type adenosine triphosphatase^21^. This interaction is mediated by the C-terminal MIM on CHMP2B and a Microtubule Interacting and Trafficking (MIT) domain on VPS4. We indeed found that PDpep1.3 inhibits the CHMP2B-VPS4 interaction (Fig. S1D). However, we also noted that many CHMP proteins can self-interact and have been reported to multimerize, which may contribute to their function^30^. In particular, CHMP2B can self-interact^30^ and is thought to polymerize at the budding of membranes during endosome formation^31^. We therefore examined whether PDpep1.3 binds to CHMP2B. By performing co-immunoprecipitation studies, we found that PDpep1.3 and CHMP2B form a complex in cells (Fig. 1D). Further, we observed that they interact directly in vitro by testing FITC-labeled PDpep1.3 peptide against purified recombinant CHMP2B in fluorescence polarization (FP) binding assays (Fig. 1E). In these assays, FITC is excited with plane-polarized light and, when it binds a larger molecule such as CHMP2B, it rotates more slowly and the emitted light remains largely polarized. To test whether the effect of PDpep1.3 on cell survival was mediated through CHMP2B or VPS4, we performed shRNA knockdown of each. We found that the cytoprotective effect of PDpep1.3 was prevented by CHMP2B knockdown, but not by knockdown of VPS4A and/or VPS4B (Figs. 1F, S1E, S1F). We noted that the CHMP2B-VPS4 interaction, though proven to be functional, is quite weak (178 µM) even for a motif-domain interaction^21^, and our affinity measurements revealed that PDpep1.3 binds to CHMP2B much more tightly (1.8 µM) than it does to VPS4 (>100 µM) (Fig. S1G). Consistent with the poor affinity to VPS4, the sequence of PDpep1.3 differs from classic MIMs in that it lacks a C-terminal positive charge (Fig. S1C), making it a weak binder of MIT domains. We tested other MIM peptides (Fig. S1C), all of which showed markedly weaker effects than PDpep1.3 in rescuing a-syn cytotoxicity (Fig. S1H), further suggesting that it is not a MIM-MIT domain interaction that is targeted. Taken together, our results instead imply that PDpep1.3 protects against a-syn cytotoxicity by targeting alternative interactions of CHMP2B. Additionally, we note that the CHMP2B self-interaction, mediated by the PDpep1.3 sequence, is much stronger than its interaction with VPS4 and thus the C-terminus of CHMP2B may play a role in its polymerization.

### PDpep1.3 promotes degradation of a-syn

**Fig. 2:**
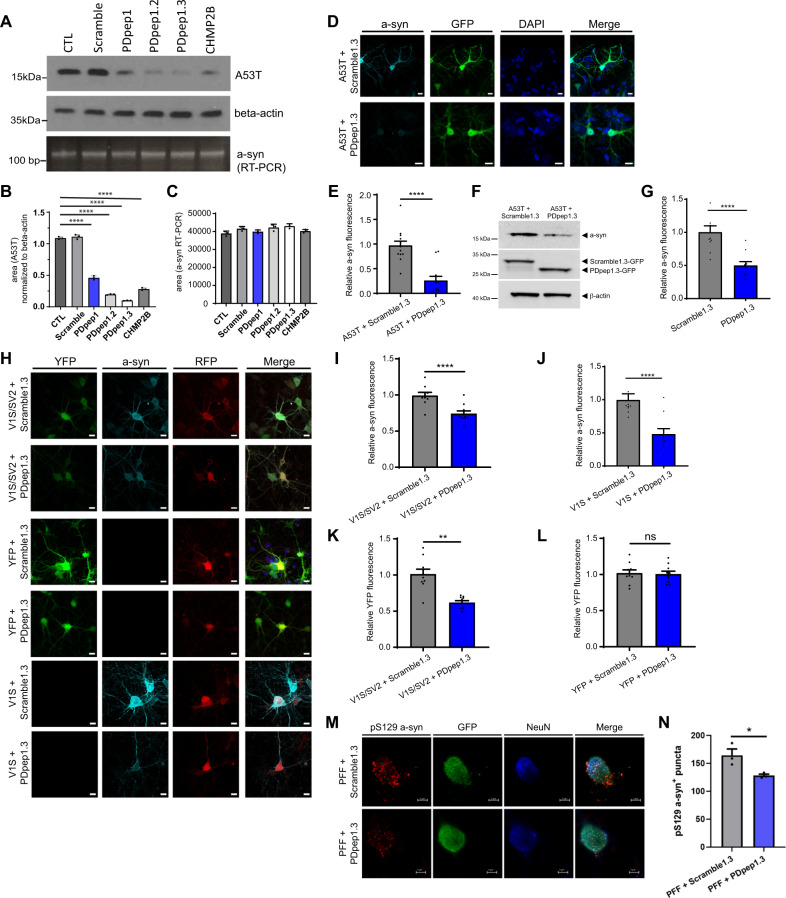
PDpep1.3 reduces a-syn levels in cell lines and primary cortical neurons. **A** Representative immunoblots of a-syn levels in HEK293 cells expressing A53T a-syn plus GFP alone (CTL), peptides, or full-length CHMP2B (top panel). Loading control was beta-actin (middle panel). RT-PCR shows no change in a-syn mRNA levels (bottom panel). **B** Quantification of A53T a-syn protein level (normalized to beta-actin) from immunoblot data represented in (**A**). Bars represent means ± s.d. (unpaired two-tailed *t*-tests; PDpep1, PDpep1.2, PDpep1.3, CHMP2B, *P* < 0.0001; *n* = 3). **C** Quantification of a-syn mRNA from RT-PCR data represented in (**A**). Bars represent means ± s.d. (*n* = 3). **D** Representative images of rat cortical neurons transduced with A53T a-syn plus Scramble1.3-GFP or PDpep1.3-GFP (scale bars = 5 µm). **E** Quantification of relative a-syn fluorescence in GFP-positive neurons. Bars represent means ± s.e.m. (two-tailed nested *t*-tests, *t*(55) = 6.545; *P* < 0.0001; *n* = 3). **F** Representative immunoblot of human a-syn (top panel) and GFP (middle panel) of lysates from cortical neurons transduced with A53T a-syn plus Scramble1.3-GFP or PDpep1.3-GFP. Loading control was beta-actin (bottom panel). **G** Quantification of relative endogenous a-syn fluorescence in GFP-positive neurons transduced with Scramble1.3-GFP or PDpep1.3-GFP. Bars represent means ± s.e.m. (two-tailed nested *t*-test, *t*(55) = 5.504; *P* < 0.0001; *n* = 3). **H** Representative images of rat cortical neurons transduced with V1S/SV2 or YFP plus Scramble1.3-RFP or PDpep1.3-RFP to assess for a-syn oligomer levels. Quantification of relative a-syn fluorescence in RFP-positive cortical neurons transduced with **I** V1S/SV2 plus Scramble1.3-RFP or PDpep1.3-RFP (two-tailed nested *t*-test, *t*(51) = 4.915; *P* < 0.0001; *n* = 3) or **J** V1S alone plus Scramble1.3-RFP or PDpep1.3-RFP (two-tailed nested *t*-test, *t*(36) = 3.468; *P* = 0.0014; *n* = 3). Quantification of relative YFP fluorescence in RFP-positive cortical neurons transduced with **K** V1S/SV2 plus Scramble1.3-RFP or PDpep1.3-RFP (two-tailed nested *t*-test, *t*(54) = 5.455; *P* = 0.0055; *n* = 3) or **L** YFP plus Scramble1.3-RFP or PDpep1.3-RFP (two-tailed nested *t*-test, *t*(56) = 0.092; *P* = 0.93; *n* = 3). **M** Representative images of cortical neurons treated with human a-syn PFFs plus Scramble1.3-GFP or PDpep1.3-GFP (scale bars = 5 μm). **N** Quantification of number of pS129 a-syn^+^ puncta per GFP-positive neuron (unpaired two-tailed *t*-test, *t*(4) = 3.099; *P* = 0.0362; *n* = 3). *P < 0.05; **P < 0.01; ****P < 0.0001; ns indicates *P* > 0.05. Source data are provided as a Source Data file.

We next sought to determine the downstream effects of PDpep1.3. Since ESCRT is involved in protein degradation with important roles in the endolysosomal pathway, we hypothesized that PDpep1.3 might promote degradation of a-syn. To test this, we examined A53T a-syn protein levels in HEK293 cells co-transfected with PDpep1 and its optimized versions, including PDpep1.3. Cells transfected with each of the peptides or with full-length CHMP2B demonstrated reduced a-syn protein levels by immunoblotting, while a scrambled control peptide had no effect (Fig. 2A, B). Levels of a-syn mRNA, measured by RT-PCR, were not different across conditions and therefore this reduction was not due to decreased transcription (Figs. 2A, C, S1I).

To ensure the effect of PDpep1.3 was not limited to immortalized cell lines, we co-transduced primary cortical neurons isolated from E17 rat embryos with adeno-associated viruses (AAVs) encoding human A53T a-syn and either GFP-tagged PDpep1.3 or Scramble1.3 control (same amino acid composition but scrambled sequence). Immunostaining of these neurons showed a significant decrease in A53T a-syn fluorescence intensity in those neurons expressing PDpep1.3 compared with Scramble1.3 (Fig. 2D, E). A corresponding reduction in total A53T a-syn protein levels with PDpep1.3 was seen by immunoblotting of the neuronal lysates (Fig. 2F). Additionally, we immunostained neurons expressing PDpep1.3 or Scramble1.3 for endogenous a-syn and found reduced fluorescence intensity with PDpep1.3 (Fig. 2G). We also examined whether PDpep1.3 could reduce WT a-syn levels in primary neurons while simultaneously measuring levels of a-syn oligomers. To this end, we transduced neurons with AAVs encoding human WT a-syn fused to the N- or C-terminal half of YFP (V1S or SV2, respectively). With co-expression of V1S and SV2 (Fig. 2H, I) or expression of V1S alone (Fig. 2H, J), a significant lowering in total a-syn fluorescence was observed with red fluorescent protein (RFP)-tagged PDpep1.3 compared with Scramble1.3. In addition, PDpep1.3 reduced YFP fluorescence from co-expressed V1S and SV2 (Fig. 2H, K) but had no effect on fluorescence of YFP alone (Fig. 2H, L). Finally, we investigated the effect of PDpep1.3 on aggregation of endogenous a-syn using a neuronal model in which addition of preformed fibrils (PFFs) synthesized from recombinant human WT a-syn seeds the formation of phosphorylated a-syn puncta within neurons^32^. We found that, compared with Scramble1.3, PDpep1.3 reduced the number of endogenous a-syn aggregates measured as puncta containing the pathological form of a-syn phosphorylated at serine 129 (pS129) (Fig. 2M, N). Thus, PDpep1.3 reduces levels of overexpressed WT or mutant a-syn protein. This is not due to effects on vector expression since mRNA levels were unchanged and a similar reduction was observed with endogenous a-syn. While PDpep1.3 reduces total a-syn levels, it also lowers the amount of a-syn oligomers and PFF-induced a-syn seeding.

### PDpep1.3 disrupts an interaction between the C-terminal region of a-syn and CHMP2B

**Fig. 3:**
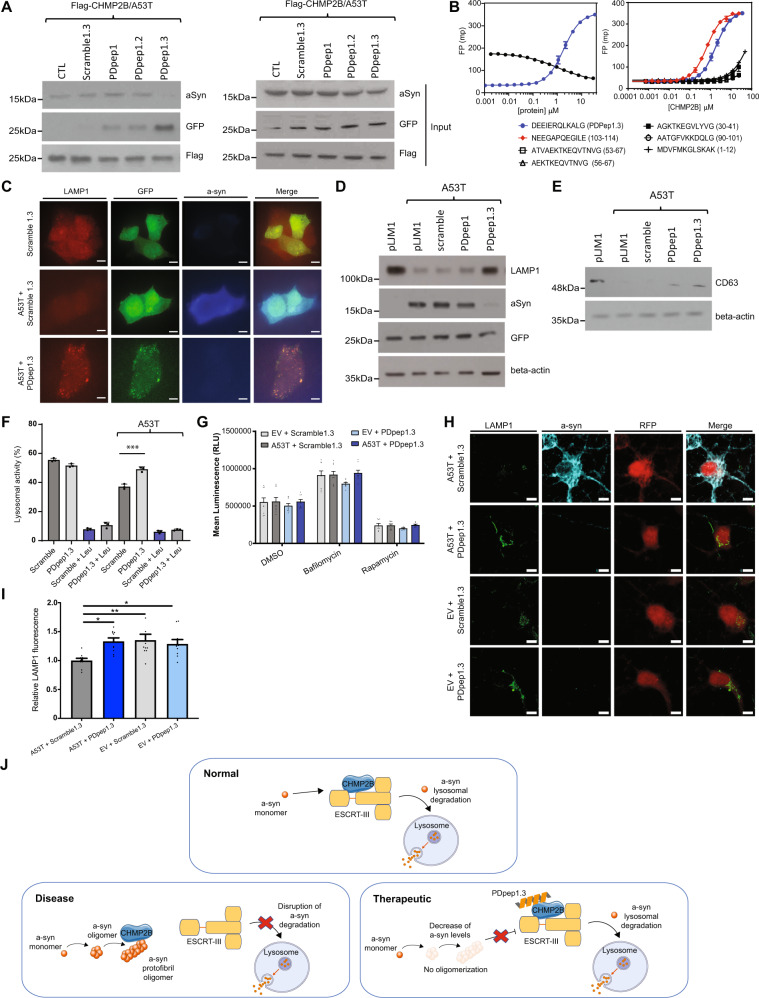
PDpep1.3 outcompetes a-syn binding to CHMP2B to enhance endolysosomal-mediated clearance of a-syn. **A** Validation of PPI disruption using co-immunoprecipitation assays. Flag-CHMP2B was immunoprecipitated in the presence of HA-tagged A53T a-syn and GFP-tagged peptides. Negative controls were GFP alone (CTL) and a GFP-tagged scrambled version of PDpep1.3 (Scramble1.3); neither markedly co-immunoprecipitated with CHMP2B nor disrupted the interaction between CHMP2B and A53T a-syn. **B** Fluorescence polarization (FP) binding assay of FITC-labeled PDpep1.3 (blue) and displacement of the fluorescent peptide with increasing concentrations of a-syn (black). Error bars represent ± s.d. of the fit (*n* = 3) (left panel). FP binding assay of FITC-labeled a-syn peptides. Error bars represent ± s.d. of the fit (*n* = 3) (right panel). **C** PDpep1.3 restores reduced LAMP1 expression due to A53T a-syn in HEK293 cells as shown by confocal micrographs (scale bars = 15 μm). Representative immunoblots of **D** LAMP1 levels or **E** CD63 levels in HEK293 cells upon co-transfection of A53T a-syn and peptides. Controls were GFP alone (pLJM1) and a GFP-tagged scrambled version of the initial peptide (scramble). Loading control was beta-actin. Experiments were done in triplicate. **F** Endolysosomal flux assay using flow cytometry in HEK293 cells co-transfected with A53T a-syn or control vector plus Scramble1.3 or PDpep1.3. Cells were treated with the lysosomal inhibitor Leupeptin (Leu) as indicated. Data represent means ± s.d. (unpaired two-tailed *t*-test, *t*(4) = 2.776; A53T+PDpep1.3, *P* = 0.0010; *n* = 3). **G** Autophagy flux assay in HEK293 cells stably expressing a luminescent LC3-HiBiT reporter co-transfected with A53T a-syn or empty vector (EV) plus Flag-Scramble1.3 or Flag-PDpep1.3. Cells were treated with the lysosomal inhibitor Bafilomycin A1 or the autophagy inducer Rapamycin as indicated. Bars represent means ± s.e.m. (*n* = 3). **H** Representative images of cortical neurons transduced with A53T a-syn or EV plus Scramble1.3-RFP or PDpep1.3-RFP (scale bars = 5 μm). **I** Quantification of LAMP1 fluorescence in RFP-positive neurons. Bars represent means ± s.e.m. (nested one-way ANOVA, *F*(3, 102) = 4.430; *P* = 0.0057; *n* = 3). **J** The proposed mechanism of PDpep1.3 is that the peptide disrupts the a-syn-CHMP2B interaction to restore degradation of a-syn by the endolysosomal pathway (created using Servier Medical Art at https://smart.servier.com/). *P < 0.05; **P < 0.01; ***P < 0.001. Source data are provided as a Source Data file.

Increased levels of a-syn have been reported to impair ESCRT and the endolysosomal pathway^29^, but the exact mechanism of this impairment remains unknown. It has been suggested that a-syn may exert an effect on the endolysosomal pathway through CHMP2B. We hypothesized that this could be mediated by a direct interaction between a-syn and CHMP2B. These proteins were previously found to co-immunoprecipitate and co-localize in transgenic a-syn mouse brains and human brains with Lewy bodies^29^. We performed co-immunoprecipitation experiments using lysates from HEK293 cells overexpressing A53T a-syn and CHMP2B and similarly showed an interaction between the two proteins. PDpep1.3 disrupted this a-syn-CHMP2B interaction in HEK293 cells (Figs. 3A, S2A, S2B, S2C). We also detected an interaction between endogenous WT a-syn and CHMP2B in naïve rat cortical neurons by a proximity ligation assay (PLA) in which oligonucleotide-hybridized antibodies generate a signal to indicate close PPIs in situ (Fig. S2D). With expression of PDpep1.3, we found a reduction of the interaction between endogenous WT a-syn and CHMP2B compared with Scramble1.3, as shown by lowered PLA signal (Fig. S2E, S2F). These results imply that a-syn and CHMP2B form a complex in neurons and that the amount of endogenous CHMP2B in complex with a-syn can be reduced by PDpep1.3.

To determine whether the a-syn-CHMP2B complex involves a direct interaction between the two proteins, we performed FP binding assays using purified recombinant WT a-syn and CHMP2B (Fig. 3B). We found that full-length a-syn could displace binding of FITC-labeled PDpep1.3 to CHMP2B. Further, we tested several a-syn peptide fragments in FP binding assays, and we mapped the interaction site to amino acids 103-114 of a-syn (NEEGAPQEGILE) (Fig. 3B). This a-syn peptide fragment directly interacted with CHMP2B in vitro with an affinity of 0.64 µM, similar to PDpep1.3. We further confirmed this finding using nuclear magnetic resonance (NMR) to demonstrate that CHMP2B directly interacts with the C-terminus of a-syn (Fig. S3A).

The C-terminal region of a-syn that binds to CHMP2B is exposed in structures of a-syn oligomers (Fig. S3B)^33^. Thus, higher levels of a-syn along with increased a-syn oligomerization could result in sequestration of CHMP2B, thereby leading to a disruption of ESCRT-III. We indeed confirmed that a-syn oligomers directly bind to CHMP2B at comparable affinity to monomers (Fig. S3C). Consistent with this, we found that addition of full-length CHMP2B reduces a-syn levels (Fig. S3D) and rescues a-syn cytotoxicity in HEK293 cells (Fig. S3E). Addition of a truncated version of CHMP2B (amino acids 1–199), in which the MIM is removed, also rescues toxicity, while a shorter truncated version (amino acids 1–178), which has been implicated in FTD^24^, actually increased toxicity (Fig. S3D, S3E). Moreover, we expressed a truncated version of A53T a-syn lacking amino acids 103–140 in neurons which was HA-tagged at its N-terminus. We observed that, compared with Scramble1.3, PDpep1.3 did not reduce levels of this truncated a-syn measured by HA immunostaining (Fig. S3F, S3G). Therefore, the a-syn-CHMP2B interaction is necessary for the a-syn lowering effects of PDpep1.3.

### PDpep1.3 rescues endolysosomal activity disrupted by a-syn

Since both CHMP2B and a-syn can influence the endolysosomal pathway, we sought to assess whether PDpep1.3 exerts its effects by modulating this pathway. We first examined endolysosomal markers in HEK293 cells overexpressing A53T a-syn. Lysosomal-Associated Membrane Protein 1 (LAMP1) is a marker of late endosomes and lysosomes that is decreased with accumulation of a-syn in brains of PD patients and rodent models^34^. Consistent with this, we found a decrease in LAMP1 levels with overexpression of A53T a-syn in HEK293 cells (Fig. 3C, D). LAMP1 levels were restored upon co-expression of PDpep1.3. If PDpep1.3 exerts this effect through disruption of the a-syn-CHMP2B interaction, mutations that prevent the interaction would be expected to have a similar effect. To test this, we made several a-syn mutants with mutations in the CHMP2B binding region. Indeed, we found that a-syn with these mutations had less of an effect on LAMP1 levels or a-syn cytotoxicity (Fig. S4A). We then assessed the effect of PDpep1.3 on CD63, a MVB membrane protein, which is a more specific marker of late endosomes^35^. Expression of A53T a-syn was associated with a substantial loss of CD63, consistent with disruption of MVB formation within the endolysosomal pathway (Fig. 3E). Co-expression with PDpep1.3, but not with Scramble1.3 control, resulted in the return of CD63.

Next, to test whether PDpep1.3 affects flux via the endolysosomal pathway, we applied an extracellular substrate that can act as endocytic cargo and be taken up by HEK293 cells. Within an endolysosomal vesicle, the substrate is degraded, and a fluorescent signal is generated that is proportional to lysosomal activity. As expected, we found that A53T a-syn reduced the fluorescent signal, consistent with impaired endolysosomal flux (Fig. 3F). However, co-expression with PDpep1.3 returned the fluorescent signal to baseline (Fig. 3F), suggesting rescue of endolysosomal pathway activity. To examine whether the degradation pathway disrupted by a-syn and rescued by PDpep1.3 also involves autophagy, we used an autophagy flux assay in which HEK293 cells express the autophagy-related microtubule-associated proteins 1 A/1B light chain 3B, or LC3, fused to a luminescent reporter. An increase in autophagic flux accelerates the degradation of the LC3 reporter, resulting in decreased LC3 reporter levels and luminescent signal, which we demonstrated with rapamycin (Fig. 3G). Conversely, inhibition of autophagy increases LC3 reporter levels and luminescent signal, which we showed with bafilomycin A1. We found that neither A53T a-syn nor PDpep1.3 altered autophagic flux in this system (Fig. 3G). Therefore, the effect of PDpep1.3 is instead likely mediated by the endolysosomal pathway.

Finally, to assess if PDpep1.3 impacts the endolysosomal pathway in neurons, we examined LAMP1 in rat cortical neurons transduced by AAV-A53T a-syn or empty AAV vector as control. Similar to HEK293 cells (Fig. 3C, D), neurons expressing A53T a-syn demonstrated decreased levels of LAMP1 and PDpep1.3 restored LAMP1 levels (Fig. 3H, I). We also examined Rab7, a small GTPase that plays a central role in the Rab cascade of the endolysosomal pathway^36,37^. Rab7 is increased with accumulation of a-syn in PD cell models^38,39^ and in human brains with Lewy bodies^40^. Consistent with this, we found an increase in Rab7 levels with overexpression of A53T a-syn in rat cortical neurons which was normalized upon co-expression of PDpep1.3 (Fig. S4B, S4C). Similarly, A53T a-syn expression was associated with increases in the lysosomal proteases, cathepsin B (Fig. S4D, S4E) and cathepsin D (Fig. S4F, S4G), which were reduced to control levels by PDpep1.3. Thus, we concluded that PDpep1.3 can normalize the changes in endolysosomal markers induced by A53T a-syn in neurons.

Based on all the above findings, we propose that accumulation and oligomerization of a-syn lead to a feedback loop in which a-syn initially inhibits protein degradation by disrupting ESCRT via CHMP2B binding (Fig. 3J). Both late endosomes and lysosomes are disrupted, thus impairing endolysosomal-mediated protein degradation. While a-syn can be cleared from the cell via a number of ways^41^, lysosomes play a key role in a-syn degradation^42^. Thus, disruption of the endolysosomal pathway increases a-syn levels which, in turn, further exacerbates dysfunction of the pathway^43^. We propose that this feedback cycle can be broken by PDpep1.3, which disrupts the a-syn-CHMP2B interaction, releasing CHMP2B to restore degradation of a-syn by endolysosomal activity (Fig. 3J).

### PDpep1.3 reduces a-syn-mediated neurodegeneration in vivo

Overexpression of WT or mutant a-syn in dopaminergic neurons causes degeneration of neurites and eventual loss of soma in several animal models^44^. We used such models to examine whether treatment with PDpep1.3 could attenuate a-syn-mediated neurodegeneration in vivo.

**Fig. 4:**
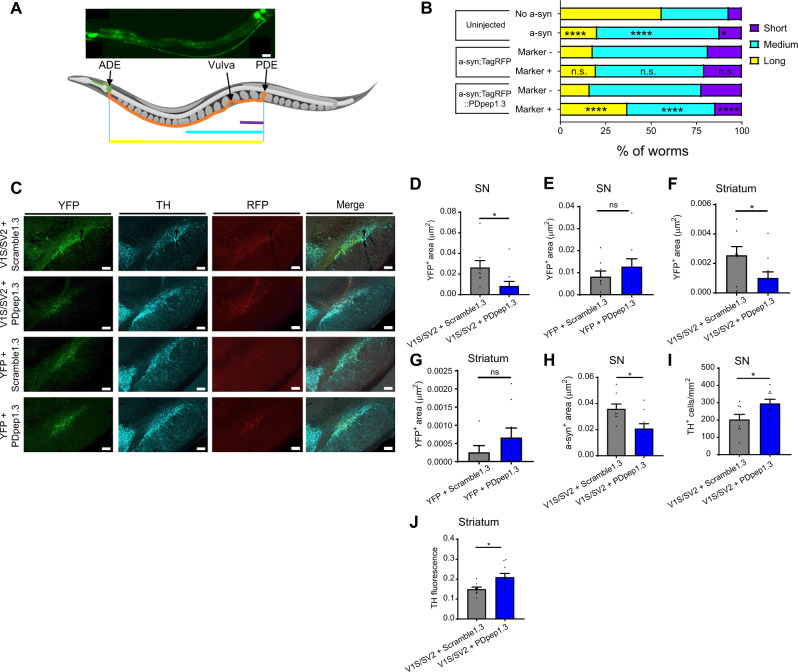
PDpep1.3 reduces a-syn-mediated neurodegeneration in *C. elegans* and an a-syn oligomer rat model. **A** Representative image of an adult *C. elegans* with PDE neuron visualized using *dat-1p::gfp*(*egIs1*) (scale bar = 20 μm) (top panel). Neurite length for each PDE neuron was categorized as: Short (does not extend past the vulva; purple), Medium (extends past the vulva but not beyond halfway to ADE neuron; cyan), or Long (extends to ADE neuron; yellow) (created using an image from somersault1824 at https://somersault1824.gumroad.com/). **B** Frequencies of PDE neurite lengths were compared for *C. elegans* expressing no a-syn versus a-syn alone (Pearson Chi-Square test, *χ*^*2*^(2, 1252) = 169.0; *P* < 0.0001), a-syn with RFP (a-syn;TagRFP Marker+) versus a-syn without RFP (a-syn;TagRFP Marker-) (Pearson Chi-Square test, *χ*^*2*^(2, 910) = 1.332; *P* = 0.5137), and a-syn with RFP-PDpep1.3 (a-syn;TagRFP::PDpep1.3 Marker+) versus a-syn without RFP-PDpep1.3 (a-syn;TagRFP::PDpep1.3 Marker-) (Pearson Chi-Square test, *χ*^*2*^(2, 1528) =  86.1729; *P* < 0.0001). **C** Representative images of native YFP and RFP fluorescence and immunostaining with anti-tyrosine hydroxylase (TH) antibody in substantia nigra (SN) of rats injected with V1S/SV2 or YFP plus Scramble1.3-RFP or PDpep1.3-RFP (scale bars = 200 μm). Quantification of YFP^+^ area in SN of rats injected with **D** V1S/SV2 (unpaired two-tailed *t*-test, *t*(16) = 2.317; *P* = 0.0341; Scramble1.3, *n* = 8 rats; PDpep1.3, *n* = 10 rats) or **E** full-length YFP (unpaired two-tailed *t*-test, *t*(15) = 1.003; *P* = 0.3317; Scramble1.3, *n* = 8 rats; PDpep1.3, *n* = 9 rats). Quantification of YFP^+^ area in striatum of rats injected with **F** V1S/SV2 (unpaired two-tailed *t*-test, *t*(16) = 2.193; *P* = 0.0434; Scramble1.3, *n* = 8 rats; PDpep1.3, *n* = 10 rats) or **G** full-length YFP (unpaired two-tailed *t*-test, *t*(13) = 1.140; *P* = 0.2749; Scramble1.3, *n* = 6 rats; PDpep1.3, *n* = 9 rats). **H** Quantification of a-syn^+^ area in SN (unpaired two-tailed *t*-test, *t*(16) = 2.843; *P* = 0.0118; Scramble1.3, *n* = 8 rats; PDpep1.3, *n* = 10 rats), **I** TH^+^ cell counts in SN (unpaired two-tailed *t*-test, *t*(15) = 2.421; *P* = 0.0286; Scramble1.3, *n* = 8 rats; PDpep1.3, *n* = 9 rats), and **J** TH fluorescence in striatum (unpaired two-tailed *t*-test, *t*(16) = 2.664; *P* = 0.0170; Scramble1.3, *n* = 8 rats; PDpep1.3, *n* = 10 rats) at 6 weeks post-injection of V1S/SV2 plus Scramble1.3-RFP or PDpep1.3-RFP. Bars represent means ± s.e.m. *P < 0.05; ****P < 0.0001; ns indicates *P* > 0.05. Source data are provided as a Source Data file.

First, we tested PDpep1.3 in *C. elegans* (Fig. 4A) using a version of the peptide based on the nematode amino acid sequence of CHMP2B. Overexpression of mutant A30P human a-syn resulted in significant neurite shortening of the posterior deirid (PDE) dopaminergic neurons, which was partially rescued by co-expression of RFP-tagged PDpep1.3, but not RFP alone (Fig. 4B).

Second, we determined the effect of PDpep1.3 on a-syn oligomerization in an AAV vector-based rat model by directly co-injecting AAV-V1S and AAV-SV2 (a-syn fused to N- or C-terminal halves of YFP, respectively; see above) in the substantia nigra (SN), as we have done previously^16^, with AAVs that express PDpep1.3 or Scramble1.3. At 6 weeks post-injection, we observed a significant reduction in a-syn oligomer levels, measured by YFP positive area, in dopaminergic neurons in the SN (Fig. 4C–E) and their axonal projections in the striatum (Fig. 4F, G) of rats that received PDpep1.3 compared with Scramble1.3. When we measured YFP fluorescence intensity in SN cells positive for both tyrosine hydroxylase (TH) (i.e., dopaminergic neurons) and RFP (i.e., cells expressing peptide), we also found that dopaminergic neurons expressing PDpep1.3 had a lower mean fluorescence intensity (0.063 ± 0.0167 relative fluorescence units (RFU)) than those expressing Scramble1.3 (0.154 ± 0.034 RFU) (*n* = 8–9 rats/group, one-way ANOVA, *P* < 0.05). These findings with PDpep1.3 were associated with a decrease in total a-syn positive area in the SN (Fig. 4H), as well as an increase in surviving dopaminergic neurons in the SN (Fig. 4C, I) and their terminals in the striatum (Figs. 4J, S5A), quantified using TH as a dopaminergic marker. In this model, overexpression of human WT a-syn is associated with accumulation of pS129 a-syn in the striatum measured as pS129 fluorescence intensity (Fig. S5B). We found that, compared with Scramble1.3, PDpep1.3 reduced pS129 a-syn accumulation in the striatum (Fig. S5B, S5C, S5D).

**Fig. 5:**
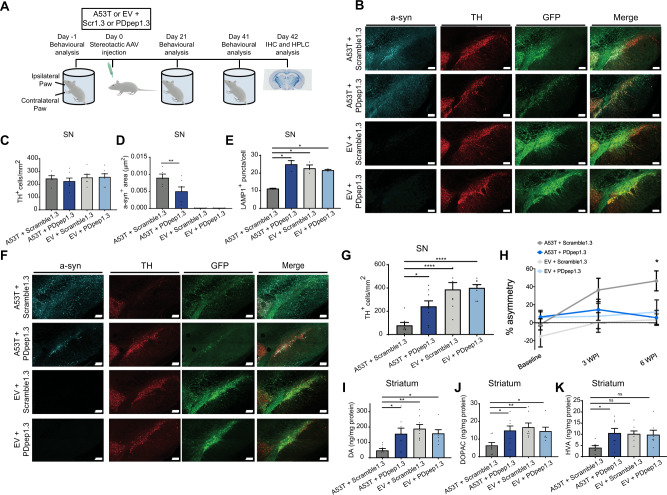
PDpep1.3 reduces a-syn levels and a-syn-mediated neurodegeneration in a preclinical rat model of PD. **A** Experimental design for testing PDpep1.3 versus Scramble1.3 in AAV-A53T a-syn rat model (created using an image from Biorender at http://www.biorender.com/). **B** Representative images of immunostaining (with anti-a-syn or anti-TH antibodies) and native GFP fluorescence in substantia nigra (SN) of rats injected with low titer A53T a-syn or empty vector (EV) plus Scramble1.3-GFP or PDpep1.3-GFP (scale bars = 200 μm). **C** TH^+^ cell counts (A53T+Scramble1.3, *n* = 5 rats; A53T+PDpep1.3, *n* = 7 rats; EV+Scramble1.3, *n* = 6 rats; EV+PDpep1.3, *n* = 7 rats), **D** quantification of a-syn^+^ area (one-way ANOVA, *F*(3, 21) = 24.89; *P* < 0.0001 followed by Dunnett’s post-test; A53T+Scramble1.3, *n* = 5 rats; A53T+PDpep1.3, *n* = 7 rats; EV+Scramble1.3, *n* = 6 rats; EV+PDpep1.3, *n* = 7 rats), and **E** LAMP1^+^ puncta counts in SN at 6 weeks post-injection of low titer A53T a-syn or EV plus Scramble1.3-GFP or PDpep1.3-GFP (nested one-way ANOVA, *F*(3, 8) = 5.780; *P* = 0.0211 followed by Dunnett’s post-test; *n* = 3 rats per group). **F** Representative images of immunostaining (with anti-a-syn or anti-TH antibodies) and native GFP fluorescence in SN of rats injected with high titer A53T a-syn or EV plus Scramble1.3-GFP or PDpep1.3-GFP (scale bars = 200 µm). **G** TH^+^ cell counts in SN at 6 weeks post-injection of high titer A53T a-syn or EV plus Scramble1.3-GFP or PDpep1.3-GFP (one-way ANOVA, *F*(3, 28) = 12.82; *P* < 0.0001 followed by Dunnett’s post-test; *n* = 8 rats per group). **H** Forelimb asymmetry in cylinder test at baseline (prior to injection), 3 weeks post-injection (WPI), and 6 WPI (repeated measures ANOVA, *F*(1.635, 37.61) = 5.185; *P* = 0.0147 followed by Tukey’s post-test; A53T+Scramble1.3, *n* = 7 rats; A53T+PDpep1.3, *n* = 7 rats; EV+Scramble1.3, *n* = 8 rats; EV+PDpep1.3, *n* = 7 rats). Quantification of **I** dopamine (one-way ANOVA, *F*(3, 27) = 5.659; *P* = 0.0038 followed by Dunnett’s post-test; A53T+Scramble1.3, *n* = 8 rats; A53T+PDpep1.3, *n* = 8 rats; EV+Scramble1.3, *n* = 8 rats; EV+PDpep1.3, *n* = 7 rats), **J** DOPAC (one-way ANOVA, *F*(3, 27) = 4.434; *P* = 0.0117 followed by Dunnett’s post-test; A53T+Scramble1.3, *n* = 8 rats; A53T+PDpep1.3, *n* = 8 rats; EV+Scramble1.3, *n* = 8 rats; EV+PDpep1.3, *n* = 7 rats), and **K** HVA (one-way ANOVA, *F*(3, 26) = 3.400; *P* = 0.0326 followed by Dunnett’s post-test; A53T+Scramble1.3, *n* = 7 rats; A53T+PDpep1.3, *n* = 8 rats; EV+Scramble1.3, *n* = 8 rats; EV+PDpep1.3, *n* = 7 rats) in striatum. Data in all graphs represent means ± s.e.m. *P < 0.05; **P < 0.01; ****P < 0.0001; ns indicates *P* > 0.05. Source data are provided as a Source Data file.

Third, we used a preclinical rat model of PD in which SN degeneration is induced by AAV vector-mediated expression of human mutant A53T a-syn^18,45–48^ (Fig. 5A). Since A53T a-syn overexpression results in substantial loss of SN neurons in this model, thus making it difficult to assess for reduction in a-syn levels not due to neuronal loss, we initially limited A53T a-syn overexpression by using a lower titer of AAV-A53T a-syn that did not result in overt neurodegeneration^49^ (Fig. 5B, C). We found a significant reduction in total a-syn positive area in the SN of PDpep1.3 treated animals compared with Scramble1.3 (Fig. 5B, D). When we measured a-syn fluorescence intensity in cells positive for both TH (i.e., dopaminergic neurons) and GFP (i.e., cells expressing peptide), we also found that dopaminergic neurons expressing PDpep1.3 had a lower mean fluorescence intensity (0.335 ± 0.025 RFU) than those expressing Scramble1.3 (0.399 ± 0.015 RFU) (*n* = 5–7 rats/group, one-way ANOVA, *P* < 0.05). Furthermore, overexpression of A53T a-syn was associated with a reduction in LAMP1 positive puncta in the SN which was restored with PDpep1.3 (Figs. 5E, S5E). We then used a higher titer of AAV-A53T a-syn which resulted in significant dopaminergic cell death in the SN at 6 weeks post-injection (Fig. 5F, G). This neurodegeneration was mitigated by treatment with PDpep1.3; the remaining number of surviving dopaminergic neurons was not different from animals injected with empty AAV vector instead of A53T a-syn and was significantly greater than treatment with Scramble1.3 (Fig. 5F, G). Forelimb asymmetry in the cylinder test is a behavioral impairment associated with SN degeneration which models bradykinesia, the cardinal motor abnormality in PD^49^. Rats injected with A53T a-syn and Scramble1.3 displayed significant asymmetry in forelimb use at 6 weeks, whereas the behavior of those injected with A53T a-syn and PDpep1.3 was comparable to controls (Fig. 5H). Consistent with mitigation of dopaminergic neuron loss by PDpep1.3, striatal levels of dopamine (Fig. 5I) and its metabolites, 3,4-dihydroxyphenylacetic acid (DOPAC) (Fig. 5J) and homovanillic acid (HVA) (Fig. 5K), were restored compared with the Scramble1.3 condition.

### PDpep1.3 reduces a-syn accumulation in human cells with PD-associated mutations

**Fig. 6:**
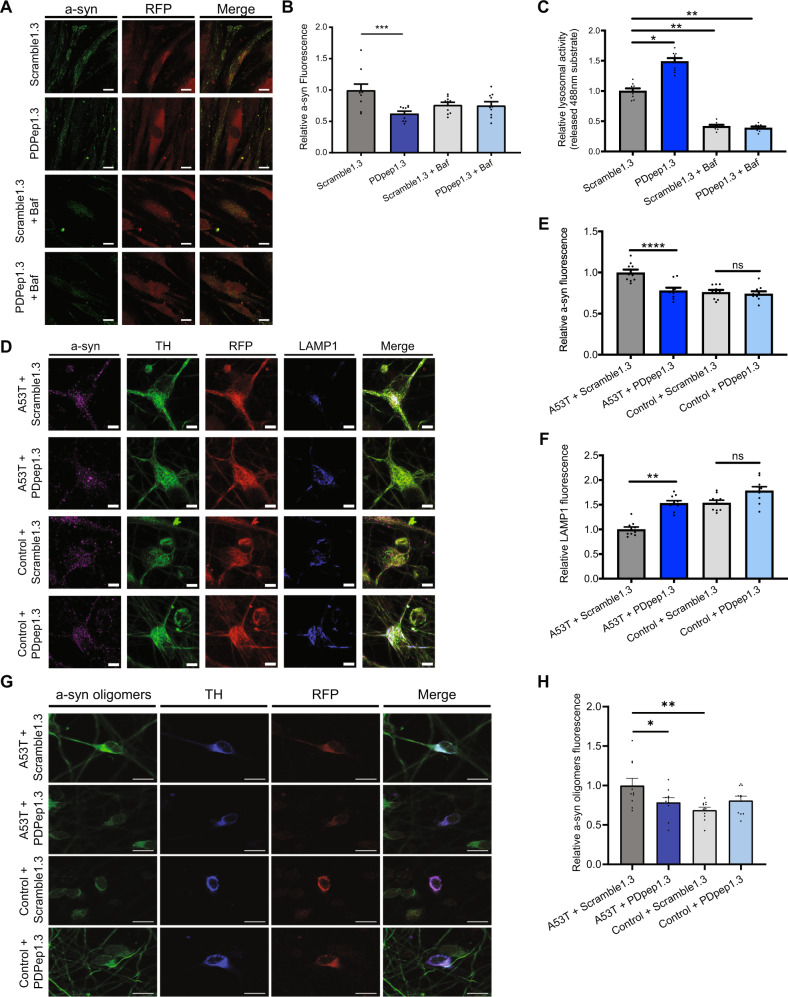
PDpep1.3 reduces endogenous a-syn levels and rescues lysosomal activity in human PD cell models. **A** Representative images of fibroblasts from a PD patient with *SNCA* triplication transduced with Scramble1.3-RFP or PDpep1.3-RFP (scale bars = 10 µm). **B** Quantification of relative a-syn fluorescence in RFP-positive fibroblasts. Bars represent means ± s.e.m. (one-way ANOVA, *F*(3, 36) = 6.159; *P* = 0.0017 followed by Dunnett’s post-test; *n* = 3). **C** Endolysosomal flux assay using confocal microscopy in *SNCA* triplication fibroblasts transduced with AAV-Scramble1.3-RFP or AAV-PDpep1.3-RFP. Cells were treated as indicated with Bafilomycin A1 (Baf). Bars represent means ± s.e.m. (nested one-way ANOVA, *F*(3, 10) =  = 23.70; *P* < 0.0001 followed by Dunnett’s post-test; *n* = 3). **D** Representative images of human iPSC-derived dopaminergic neurons with A53T a-syn mutation or isogenic controls without a-syn mutation. These iPSC-derived dopaminergic neurons were transduced with Scramble1.3-RFP or PDpep1.3-RFP and immunostained with anti-a-syn, anti-TH, or anti-LAMP1 antibodies (scale bars = 5 μm). **E** Quantification of relative a-syn fluorescence intensity in RFP-positive A53T mutant and control iPSC-derived dopaminergic neurons. Bars represent means ± s.e.m. (two-tailed nested *t*-tests; A53T, *t*(78) = 5.096, *P* < 0.001; Control, *t*(75) = 0.2708, *P* = 0.80; *n* = 3). **F** Quantification of relative LAMP1 fluorescence intensity in RFP-positive A53T mutant and control iPSC-derived dopaminergic neurons. Bars represent means ± s.e.m. (two-tailed nested *t*-tests; A53T, *t*(55) = 4.748, *P* = 0.009; Control, *t*(54) = 0.7178, *P* = 0.51; *n* = 3). **G** Representative images of human iPSC-derived dopaminergic neurons with A53T a-syn mutation or isogenic controls without a-syn mutation. These iPSC-derived dopaminergic neurons were transduced with Scramble1.3-RFP or PDpep1.3-RFP and immunostained with anti-a-syn oligomer or anti-TH antibodies. **H** Quantification of relative a-syn oligomer fluorescence intensity in RFP-positive A53T mutant and control iPSC-derived dopaminergic neurons. Bars represent means ± s.e.m. (nested one-way ANOVA, *F*(3, 115) = 4.715, *P* = 0.0039 followed by Dunnett’s post-test, *n* = 3).*P < 0.05; **P < 0.01; ***P < 0.001; ****P < 0.0001; ns indicates *P* > 0.05. Source data are provided as a Source Data file.

To investigate the effects of PDpep1.3 on endogenous a-syn in disease-relevant human models, we used cells with a-syn gene (*SNCA*) mutations known to cause PD. An autosomal dominant form of PD is caused by triplication mutation of *SNCA* with doubling in the effective load of the WT gene peripherally and in the brain^50^. We cultured skin fibroblasts from a PD patient with *SNCA* triplication and transduced them with AAVs to express RFP-tagged peptides. We found that PDpep1.3 reduced a-syn fluorescence compared with Scramble1.3 control (Fig. 6A, B). This reduction in a-syn levels was associated with increased endolysosomal flux as measured using the assay described above (Fig. 6C). PDpep1.3 could not reduce a-syn levels in the presence of Bafilomycin A1, which inhibits the vacuolar-type proton pump responsible for acidification of lysosomes, suggesting that PDpep3.1 acts further upstream in the endolysosomal pathway.

We also derived dopaminergic neurons from induced pluripotent stem cells (iPSCs) harboring the A53T a-syn mutation and from isogenic iPSCs in which this mutation was absent. iPSC-derived dopaminergic neurons from people with genetic or idiopathic PD have been observed to accumulate a-syn protein^51–53^. Under control conditions (i.e., expression of Scramble1.3 peptide), we also observed a-syn accumulation in A53T a-syn iPSC-derived dopaminergic neurons when compared with isogenic neurons (Fig. 6D, E). Further, we used an antibody that has a high binding affinity for oligomeric a-syn and recognizes early soluble oligomers and late fibrils of a-syn (Syn-O2)^54^. This antibody detects these a-syn conformations in cerebrospinal fluid^55^ and brain tissue^54^ from PD patients. Levels of these a-syn conformations were increased in A53T a-syn neurons (Fig. 6G, H). Further, LAMP1 levels were reduced in A53T a-syn neurons (Fig. 6D, F). We found that expression of PDpep1.3 in A53T a-syn neurons returned levels of a-syn, including oligomeric a-syn, as well as LAMP1 levels to those of isogenic controls (Fig. 6D–H). Taken together, these data suggest that, in addition to its neuroprotective effects in animal models, PDpep1.3 is efficacious at reducing a-syn levels and restoring the endolysosomal pathway in human PD models.

## Discussion

Our results show that a-syn inhibits the endolysosomal pathway via the interaction of its C-terminal region with CHMP2B. Buildup of a-syn levels will lead to more sequestration of CHMP2B by mass action, as well as to oligomerization of a-syn. We show that a-syn oligomers retain affinity to CHMP2B and thus contribute to reduction of endolysosomal function. Moreover, this reduced endolysosomal function will likely lead to reduced degradation of a-syn itself, thus resulting in further accumulation, which in turn leads to further endolysosomal disruption. Our findings thus illustrate a potential mechanism by which WT or mutant a-syn accumulation and oligomerization can lead to severe failure of the cellular machinery.

The optimized peptide derived from our proteomic screens, PDpep1.3, directly disrupts the a-syn-CHMP2B interaction, breaking the feedback loop and thereby restoring ESCRT function and lysosomal degradation. In neurons, PDpep1.3 normalizes derangements in the endolysosomal pathway caused by a-syn as demonstrated by a return to baseline levels of LAMP1, Rab7, cathepsin B, and cathepsin D. Although others have shown that overexpression of some of these proteins may rescue a-syn toxicity^56,57^ in the context of a-syn accumulation, PDpep1.3 is reversing the deleterious effect of a-syn on this pathway. The net overall effect of PDpep1.3 is an increase in endolysosomal flux and consequently enhanced clearance of a-syn, including a-syn oligomers. While there have been several studies promoting biomimetic peptides inhibiting a-syn aggregation, PDpep1.3 is notable in that it operates in a distinct manner, specifically breaking the aforementioned feedback loop.

We have demonstrated the effectiveness of PDpep1.3 in promoting clearance of overexpressed a-syn both in vitro and in vivo, and we have shown that expression of PDpep1.3 can reduce dopaminergic cell death in preclinical models of PD. We have also shown PDpep1.3 to be efficacious at inhibiting a-syn accumulation and restoring the endolysosomal pathway in human PD models. This has potential important therapeutic implications since a peptide, such as PDpep1.3, that reduces overall a-syn protein levels by facilitating degradation will circumvent many of the challenges faced by current approaches aiming to target specific a-syn conformations, including peptides or small molecules directly targeting a-syn aggregates. Furthermore, PDpep1.3 demonstrated efficacy in models expressing mutant a-syn, but also WT a-syn which accumulates in idiopathic PD. Thus, targeting this pathogenic interaction between a-syn and CHMP2B may hold promise as a disease-modifying therapeutic strategy for the treatment of PD.

Challenges of course remain for the clinical application of PDpep1.3 including the penetration of the blood brain barrier (BBB), as well as issues related to properties of peptides, such as cell permeability and low serum stability. We note that solutions for all these challenges do exist including, but not limited to, modified peptides that can cross the BBB and cell membrane, as well as peptides with exquisite serum stability^3,58,59^. More recently, engineered AAVs have been developed which effectively cross the BBB and have selective tropism for specific cell types in the central nervous system and thus could be used to deliver DNA encoding the peptide^60^. Neurosurgical techniques have also evolved in parallel to provide other potential options for delivery of peptides, such as PDpep1.3, including direct intraparenchymal delivery of recombinant peptide through catheter infusion systems^61^ or packaged in current generation gene therapy vectors delivered with real time MRI guidance^62^. Further, developments using low-intensity focused ultrasound to transiently open the BBB in a spatially conformal manner might allow for delivery of therapeutic peptides, such as PDpep1.3^63^. Safety and feasibility of this technology is already being testing in people with PD^64^.

Further investigation is warranted to fully explore potential limitations of treatment with PDpep1.3. As with other therapeutics in development for neurological diseases, a detailed determination of the spatial and temporal kinetics within neurons will be required to fully understand target engagement. This will also require mapping out when in the feed forward cycle of endolysosomal dysfunction the intervention will be most efficacious. In addition, off-target effects will need to be defined to anticipate adverse events with a focus on how PDpep1.3 engages these pathways in the disease state. Our analyses in iPSC-derived neurons as human models suggest that engaging this pathway holds promise, but further work will be required in other iPSC lines to ensure our findings are generalizable.

## Methods

### Cell cultures

HEK293T cells (CRL-3216) and HEK293 cells (CRL-1573) were obtained from ATCC. Primary neurons were cultured from embryos (E17) removed from pregnant Sprague-Dawley rats (purchased from Envigo). *SNCA* triplication fibroblasts were obtained from National Institute of Neurological Disorders and Stroke (NINDS) Human Genetics Resource Center DNA and Cell Line Repository (NINDS Repository sample number ND27760). A53T a-syn iCell Dopa neurons (catalog #R1109) and isogenic control iCell Dopa neurons (catalog #R1088) were purchased from Fujifilm Cellular Dynamics (Madison, WI, USA).

### Cloning of the lentiviral library

To identify peptide inhibitors of PPI, we used a previously designed human peptide library containing 50,549 heptamer C-terminal sequences, corresponding to 75,797 proteins, including isoforms and cleaved sequences. The oligonucleotide libraries were amplified using amplification primers (forward 5′-GGAACTAGAATTCTGCCCCCGGTGGCGGA-3′ and reverse 5′-TAGTTCCCCCGGGGCCTTTAATTGTATCGGT-3′) and cloned into pLJM1 nGFP vector using EcoRI and XmaI restriction enzymes.

### Lentiviral delivery and cell screens

HEK293T cells (ATCC, CRL-3216) were maintained in DMEM supplemented with 10% FBS and 1% pen/strep/glutamine, and the appropriate selection antibiotics when required. Lentiviruses were made in a 15 cm dish format by transfecting packaging cells (HEK293T) with a three-plasmid system (expression vector, psPAX2 and pMD2.G) and using Polyjet DNA transfection reagent (FroggaBio, SL100688.1). Viral transduction of HEK293T cells were performed with a multiplicity of infection (MOI) of 0.3. Infected cells were selected in puromycin-containing medium to eliminate uninfected cells and three aliquots of cells were collected for sampling of the initial (T0) cell population. Cells were treated with MG132 at concentrations of 0, 10, 25, or 50 µM. For the cell viability screen, surviving cells were collected and gDNA was extracted for identification of peptide inhibitors of cell toxicity. For the inhibition of a-syn aggregation screen, a protein fragment complementation assay (PCA) was used by transfecting cells with split YFP-a-syn constructs following stable expression of the peptides. Cells were sorted based on their YFP fluorescence intensities with a FACSVantage SE cell sorter (BD Bioscience) and gDNA was extracted for identification of peptides.

### Fluorescence-activated cell analysis and sorting (FACS)

Cells were harvested by trypsin treatment and centrifuged at 500 × *g* for 5 min. The pellet was resuspended in ice-cold PBS and centrifuged again. The pellet was then resuspended at a concentration of 4 × 10^6^ cells/ml in the sorting buffer, which is PBS containing 100 Kunitz DNase I/ml, 10 μg/ml propidium iodide (Sigma), and 2% FBS. The cells were then sent through a 40 μm filter to remove large clumps and loaded into either a FACScan Flow Cytometer (BD Bioscience) for cell analysis or a FACSVantage SE cell sorter (BD Bioscience) for cell sorting. The cells with positive propidium iodide staining (i.e., dead cells) were first eliminated from the analysis.

### Genomic DNA preparation and Illumina sample preparation

Genomic DNA (gDNA) from peptide expressing cells at different time-points was extracted using QIAamp DNA Blood Mini Kit. PCR amplifications of peptides from gDNA in parallel with the lentiviral plasmid library (naïve library) were performed using indexed Illumina PCR primers to incorporate both the Illumina adapter sequences and indexing sequences. Each 50 μl reaction contained 3.2 μg of template, 2× PCR buffer, 2× enhancer solution, 300 μM each dNTP, 900 nM each of Adapter A (5′-AATGATACGGCGACCACCGAAATG-GACTATCATATGCTTACCGTAACTTGAA-3′) and Adapter B (5′-CAAGCAGAA-GACGGCATACGATGTGGATGAATACTGCCATTTGTCTCGAGGTC-3′), 1 mM MgSO_4_, 3.75 units of Platinum Pfx polymerase, and water to 50 μl. The PCR reaction was performed by denaturing at 94 °C for 5 min, followed by cycling (94 °C for 30 sec, 65 °C for 30 sec, 68 °C for 30 sec) ×28 cycles, 68 °C for 5 min, then cooling to 4 °C. The resulting 244 bp product was purified by electrophoresis in 2% agarose followed by gel extraction. Peptide libraries were quantified using Quant-It assay (Invitrogen) and pooled. The insert size of the pooled library was confirmed on an Agilent Bioanalyzer High Sensitivity DNA chip (Agilent Technologies), and the size corrected concentration was determined with RT-qPCR (KAPA biosystems Illumina standards). 11.4 pM of peptide library and 0.6 pM of PhiX control library (Illumina) were denatured and loaded on a HiSeq 2000 V3 150 cycle sequencing kit, with a read length of 150 bp.

### Next-generation sequencing preprocessing

PCR primers that included the Illumina adaptor sequences were used to amplify peptide coding sequences recovered from selection and cell sorting. Results were demultiplexed, and peptide counts were tallied. After demultiplexing, only reads with an average quality Phred score of >30 were selected, and frequencies were calculated. Finally, the reads were then normalized to the total number of peptides read for that sample population, and a scalar factor was applied. In parallel, approximately 25 individual colonies were Sanger sequenced to compare to the NGS data.

### Determination of target interactions

The identification of potential targets for the active peptides was conducted by mapping the sequences to the interacting interfaces of PPIs in the PDB. Multiple sequence mismatches were allowed, and a maximum of three mismatches were applied. The mapping complexes were ranked by sequence identity and the number of GO terms enriched in PD were annotated for the proteins involved in the PPI (Table S1). The enrichment term analysis was performed using DAVID based on the list of 330 genes annotated by the Parkinson’s Disease Gene Ontology Annotation Institute at University College London. Next, we counted how many of the Parkinson’s standard GO terms were shared by the mapped proteins on each structure complex. We used this record to rank and prioritize the matches. After visual inspection of a list of ten hits, by discarding crystal packing contacts and non-significant matches, we selected the mapping of IPIQLKA to the structure of the complex of a C-terminal fragment of CHMP2B binding to the MIT domain of VPS4B (PDB 2JQK). Both proteins belong to the ESCRT-III complex and play a vital role in vesicular body formation.

### Validation of individual peptides

Oligonucleotides encoding the specific peptides were synthesized and individually cloned into the pLJM1 nGFP lentiviral vector. HEK293T cells were infected with individual constructs, and cell viability was assessed using Cell Titer-Glo luminescent assay (Promega) at 72 h post infection. Tet-off HEK293 cells stably expressing split luciferase-a-syn constructs and tet-off parent HEK293 cell lines were infected with individual constructs, and a-syn oligomer levels were estimated using a luciferase PCA.

### Cell Titer-Glo luminescent cell viability assay

Cells were trypsinized from subconfluent cultures as described earlier, suspended in culture media, and then seeded into triplicate wells of a 96-well plate (100 µl/well) at a density of 1.5 × 10^4^ cells per well with standard culture conditions of 5% CO_2_ at 37 °C. Cells were infected with lentivirus expressing peptide at a MOI of 5 for 72 h or transfected with plasmid for 72 h. Cell Titer-Glo reagent was added to each well (30 µL), according to the manufacturer’s protocol and optical density of the plate was measured at 540 and 630 nm with a standard spectrophotometer.

### Luciferase protein fragment complementation assay (PCA)

Tet-off HEK293 cells stably expressing split luciferase-a-syn constructs and tet-off parent HEK293 cell lines were maintained in DMEM supplemented with 10% FBS and 1% pen/strep/glutamine. Doxycycline at 1 ng/mL was included when inhibition of gene expression was required. Cells were trypsinized from subconfluent culture and seeded in a 96-well plate at a density of 15,000 cells per well. Cells were incubated overnight at 37 °C in 5% CO_2_. Cells were transfected with GFP-peptide plasmids. After 6 h of incubation, 20 µL of cell medium was transferred to a black flat-bottomed 96-well plate. Fifty microliters of Working solution (Pierce Gaussia-Firefly Luciferase Dual Assay Kit, Thermo Scientific, 16181) were added into each well containing cell medium. Immediately after adding the reagent, samples were read using a luminometer with a 480 nm filter.

### Flag co-immunoprecipitation

HEK293T cells were co-transfected with Flag-tagged target protein, HA-tagged source protein, and GFP-tagged peptide or GFP. Cells were lysed 48 h after transfections with radioimmune precipitation assay buffer (50 mM Tris-HCl pH 7.4, 1% Nonidet P-40, 150 mM NaCl, 1 mM EDTA, 10 mM Na_3_VO_4_, 10 mM sodium pyrophosphate, 25 mM NaF, 1× protease inhibitor mixture (Sigma)) for 30 min at 4 °C and co-immunoprecipitated with Flag beads (Clontech). The resulting immunocomplexes were analyzed by immunoblot using the appropriate antibodies. Protein samples were separated using 4–20% Mini-PROTEAN Tris-glycine gels (Bio-Rad) transferred to PVDF membranes and blocked in 5% milk containing PBS-Tween-20 (0.1%) for 1 h. PVDF membranes were then incubated with specified primary antibodies followed by incubation with horseradish peroxidase-conjugated secondary antibodies (anti-mouse HRP-linked, Cell Signaling, catalog #7076, 1:5000; anti-rabbit HRP-linked, Cell Signaling, catalog #7074, 1:5000) and detected using enhanced chemiluminescence (GE Healthcare). HA antibodies were obtained from Santa Cruz (catalog #7392, 1:2000), GFP antibodies were purchased from Abcam (catalog #ab290, 1:2000), and Flag antibodies were purchased from Sigma (catalog #A8592, 1:1000).

### RNA extraction and RT-PCR

RNA extraction was performed using the RNeasy kits from Qiagen (74106). RT-PCR was performed with Luna Universal One-Step RT-qPCR Kit from NEB (E3005S), using the Applied Biosystems 7300 real time PCR System (4406984). RT-PCR consisted of 40 cycles of 95 °C for 10 sec and 60 °C for 30 sec, and a final cycle (95 °C for 15 sec and then 60 °C) generated a dissociation curve. Primers used for a-syn RT-PCR: 5’-CGGGAACTAAGCAGTGTAGAAG-3′ (Sense), 5′-TTCTCTCAGAGCCTTGAATGTG-3′ (AntiSense).

### Purification of CHMP2B

Overexpress® C41 *E. coli* cells were transformed by heat shock with 100 ng of pET-DEST42 CHMP2B vector and plated in LB plates containing 50 µg/ml of Carbenicillin for overnight incubation at 37 °C. Transformed cells were grown in 500 ml of 2×YT media at 37 °C shaking at 220 rpm in a baffled 2 L flask. Cultures were grown until an OD of 0.6 was reached and then induced with 0.5 mM IPTG. After induction, cells were left shaking at 37 °C for a further 3 h at 220 rpm. Faster expression demonstrated reduced levels of non-specific truncations compared to overnight incubations at lower temperature. Cultures were pelleted at 3000 × *g* and pellets were resuspended in 20 ml of BugBuster® Master Mix per 500 ml culture. The lysis reactions were incubated for 20 min at 4 °C mixing in a tube rotator. CHMP2B constructs expressed as inclusion bodies were insoluble in the BugBuster mix. The lysates were spun for 20 min at 3000 × *g* to separate the inclusion bodies from the rest of the cell debris. Inclusion bodies were resuspended in 35 ml of 10 mM Phosphate pH 7.4, 150 mM NaCl, 6 M Guanidine HCl and spun at 34,000 × *g* at 4 °C for 20 min to remove lipidic contaminants. The supernatant was mixed with 5 ml of Ni-NTA resin in batch and incubated for 20 min in a tube rotator. Ni-NTA resins were pelleted by centrifugation at 270 × *g* for 5 min and the supernatant was removed. The resins were washed three times with 10 mM Phosphate pH 7.4, 150 mM NaCl, 6 M Guanidine HCl, 30 mM Imidazole and proteins were eluted in 10 mM Phosphate pH 7.4, 150 mM NaCl, 6 M Guanidine HCl, 500 mM Imidazole to a total volume of 10 ml. The eluted samples were dialyzed overnight in 5 L of 50 mM Na Acetate pH 5.5 and 0.5 mM TCEP at 4 °C with a 10 kDa cutoff dialysis membrane. CHMP2B was further purified by size exclusion chromatography with a HiLoad Superdex 16/60 S200 in an AKTA purifier. The column was pre-equilibrated in fresh 50 mM Na Acetate pH 5.5 and 0.5 mM TCEP, as CHMP2B displays greatly improved solubility in slightly acidic pH. CHMP2B eluted at the expected volume for a 26 kDa monomer. Samples were concentrated in a Amicon Ultra-15 spin concentrator to 0.5 mg/ml and stored at −80 °C. Sample purity was confirmed by SDS-PAGE and protein identity was validated by ESI Mass Spectrometry.

### Purification of VPS4B

VPS4B was expressed as a His-tagged Sumo construct in a pRSet B vector. Overexpress® C41 *E. coli* cells were transformed by heat shock with 100 ng of vector and cultures were grown as described for CHMP2B. Cultures were induced with 0.5 mM IPTG and incubated overnight at 20 °C. Cells were pelleted as described and resuspended in PBS with a cOmplete Mini, EDTA-free protease inhibitor tablet. Cells were lysed by sonication in a Branson Digital Sonifier at 20% amplitude for 5 min in 10 sec intervals between on and off sonication. The homogenized sample was spun at 34,000 × *g* at 4 °C for 20 min to remove insoluble contents. The soluble supernatant was incubated with 5 ml of Ni-NTA resin in batch for 20 min at 4 °C in a tube rotator. The resins were washed three times in PBS with 30 mM Imidazole and eluted in PBS with 300 mM Imidazole. Imidazole was removed by dialyzing twice into 5 L of PBS.

### Purification of a-syn

WT a-syn was a kind gift from Byron Caughey, PhD (Rocky Mountain Laboratories, NIH). Glycerol stocks were plated on LB plates containing 50 μg/ml of kanamycin for overnight incubation at 37 °C. Bacterial cultures were grown in 500 ml of LB media at 37 °C with shaking at 220 rpm in a baffled 2 L flask. Cultures were grown until an OD of 0.6 was reached and then induced with 0.5 mM IPTG. After induction, cells were incubated at 37 °C for a further 2.5 h shaking at 220 rpm. Cells were harvested by splitting the 500 ml culture into two 250 ml conical tubes and spinning down at 4000 × *g* for 15 min at 4 °C. Cells were resuspended in lysis buffer (50 mM KH_2_PO_4_, 400 mM NaCl, 100 mM KCl, 30 mM Imidazole, 10% v/v Glycerol, 0.5% v/v Triton-X-100), and lysed by 3 cycles of flash freezing on an ethanol/dry-ice mix for 12 mins, followed by heating in a water bath for 12 min. The resultant mixture was spun down at 4000 × *g* for 15 min at 4 °C and the supernatant was added directly to a His-spin trap (GE Healthcare) as per the manufacturer’s instructions. The eluted product was dialyzed against PBS overnight, aliquoted at a concentration of 1 mg/ml, and stored at −80 °C until ready for use.

### Fluorescence polarization assays

Fluorescence polarization assays were carried out in 384-well black non-binding plates (Greiner 781906) in a Pherastar plate reader (BMG) with a Fluorescence Polarization Module 485-520-520. All peptides were synthesized by LifeTein with N-terminal FITC moieties. Binding assays were performed in 50 mM Na Phosphate pH 5.5, 0.5 mM TCEP, 0.005% Triton-X-100^21^ or in 20 mM Na Acetate pH 5.5, 0.5 mM TCEP, 0.005% Triton-X-100 as indicated. FITC-labeled peptides were kept at a constant 50 nM concentration and CHMP2B was serially diluted 1:1 starting from 30 μM (0.5 mg/ml).

The a-syn competition assay had a constant 50 nM concentration of FITC-PDpep1.3 peptide and 1 μM of CHMP2B; a-syn was serially diluted 1:1 starting from 30 μM. Plates were incubated for 30 min at room temperature before reading. Raw FP data in millipolarization units (mp) were fitted to the following equation in Graphpad PRISM 8:

$$Y\,=\frac{B\max \cdot \,X}{Kd\,+\,X}\,+\,{{{{{{\mathrm{Background}}}}}}}$$

where *X* is the concentration of CHMP2B. The equation was fitted with and without Hill value (*h*) to check for *h* ≈ 1. As the FITC-labeled peptide is at a concentration under 10 times the *K*_*d*_, total CHMP2B concentration can be assumed to approximate free CHMP2B concentration. *B* max represents the polarization units in maximum association and Background represents the polarization units in absence of CHMP2B. *Y* is in mp.

### Adeno-associated viruses

Adeno-associated virus (AAV) of a 1/2 serotype was used to express A53T a-syn (AAV-A53T), a truncated version of A53T a-syn lacking amino acids 103–140 (Δa-syn(103–140)), and WT a-syn, fused to either the N-terminal half of YFP (AAV-V1S) or the C-terminal half of YFP (AAV-SV2), all under the control of the CAG promoter, a hybrid of the chicken beta-actin (CBA) promoter fused with the cytomegalovirus (CMV) immediate early enhancer sequence (GeneDetect Ltd.)^46^. An AAV1/2 vector lacking the A53T a-syn open reading frame was used as an empty vector control (EV) for A53T a-syn experiments and an AAV1/2 vector expressing full-length YFP was used as a control for experiments measuring a-syn oligomer formation.

### Primary neuron culture and AAV transduction

Pregnant rats (E17) of the Sprague-Dawley strain were purchased from Envigo. Embryos were surgically removed from the mothers and cortices dissected in Hanks Balanced salt solution (Gibco). The meninges were removed, and cells were dissociated using a papain dissociation system (Worthington) before being resuspended in Neurobasal medium A supplemented with antibiotic-antimycotic solution (Gibco), L-glutamine substitute (GlutaMAX™; Gibco) and factor B27 (Gibco). Cells were plated on poly-D-lysine coated glass coverslips at a density of 5 × 10^5^ cells/well, or on poly-D-lysine coated six-well cell culture plates at a density of 2 × 10^6^ cells/well, and incubated at 37 °C in 5% CO_2_ with half media changes every 3 days. Cells were transduced with A53T a-syn or EV AAVs at 2 days post-isolation at a MOI of 3000. After 3 days, media containing AAV vectors were removed. Cells were transduced with Scramble1.3 or PDpep1.3 AAVs at 2- or 5-days post-isolation at a MOI of 3000. Cells were fixed with 4% paraformaldehyde (PFA) for immunofluorescence staining or lysed for immunoblotting at 8 days post-isolation.

### Immunoblotting

Infected or transfected cells were scraped from six-well dishes and lysed with lysis buffer (50 mM Tris-HCl pH 7.4, 1% Nonidet P-40, 150 mM NaCl, 1 mM EDTA, 1× protease inhibitor mixture (Sigma)) for 30 min at 4 °C. The insoluble pellet was removed following a 10,000 rpm spin for 5 min at 4 °C. Lysates were analyzed by SDS-PAGE/immunoblot using 4–20% Mini-PROTEAN Tris-glycine gels (Bio-Rad), transferred to polyvinylidene fluoride (PVDF) membranes, and blocked in 5% milk containing PBS-Tween-20 (0.1%) for 1 h. PVDF membranes were then incubated with specified primary antibodies followed by incubation with horseradish peroxidase-conjugated secondary antibodies (anti-mouse HRP-linked, Cell Signaling, catalog #7076, 1:5000; anti-rabbit HRP-linked, Cell Signaling, catalog #7074, 1:5000) and detected using enhanced chemiluminescence (GE Healthcare). Anti-a-syn antibody (clone 42) was obtained from BD Biosciences (catalog #610787, 1:1000). Anti-LAMP1 antibody was obtained from Abcam (catalog #ab24170, 1:500). Anti-beta-actin antibody was obtained from Cell Signaling (13E5) (catalog #4970, 1:1000). Anti-CD63 antibody was obtained from R&D Systems (catalog #MAB50482-SP, 1:1000).

Infected neurons were scraped from 6-well dishes and lysed with RIPA buffer containing protease inhibitor cocktail (Roche). The Triton-X-100 soluble fraction was then separated from the insoluble pellet by centrifugation. Protein concentration was quantified using the DC protein assay (BioRad). For each condition, 20 μg of protein lysate was run on 4–15% acrylamide gels (BioRad) and subsequently transferred onto a PVDF membrane. Blots were blocked with 5% skim milk in TBS + 0.1% Tween-20 (TBS-T) for 1 h prior to incubation with primary antibody overnight at 4 °C. Blots were subsequently washed three times in TBS-T for 10 min per wash, incubated in species specific secondary antibody for 1 h at 21 °C, washed again, and then developed using ECL immunoblotting substrate (Pierce) and visualized on HyBlot CL autoradiographic film (Denville Scientific).

### Immunofluorescence staining of cells

After fixation, cells were permeabilized with 0.2% Triton-X-100 for 15 min, washed three times with PBS and then incubated with blocking solution (1% BSA, 22.52 mg/mL glycine, 0.1% Tween-20 in PBS or 0.4% saponin, 1% BSA, and 5% goat or donkey serum in PBS) for 1 h. Primary antibodies were diluted in incubation solution (1% BSA, 22.52 mg/mL glycine, 0.1% Tween-20 in PBS or 0.1% saponin, 1% BSA, and 5% goat or donkey serum in PBS) and incubated overnight at 4 °C. Following three washes with PBS, cells were next incubated with secondary antibodies diluted in incubation solution for 1 h at room temperature. Following another three washes with PBS, nuclei were counterstained with DAPI (ThermoFisher) and then coverslips were mounted on slides using Dako fluorescence mounting medium (Agilent) and sealed using clear nail varnish.

### Preformed fibril (PFF)-induced a-syn seeding

PFFs synthesized from recombinant human a-syn were added to primary neuronal cultures to seed recruitment of endogenous a-syn into pS129 a-syn^+^ aggregates^32^. Recombinant human a-syn PFFs were purchased from StressMarq Biosciences (SPR-322B). Cells were plated on poly-D-lysine coated glass coverslips in 24-well cell culture plates at a density of 5 × 10^5^ cells/well, and incubated at 37 °C in 5% CO_2_ with half media changes every 3 days. Cells were transduced with GFP-tagged Scramble1.3 or PDpep1.3 AAVs on day 2 post-isolation at a MOI of 3000. On day 8 post-isolation, cells were treated with 1.5 µg of recombinant a-syn PFFs. Prior to treatment, the PFFs were sonicated in a water bath sonicator applying the following settings: amplitude = 80, process time = 30 min, pulse on = 30 s, pulse off = 30 s, and temperature = 10 °C. The sonicated PFFs were divided into two aliquots: one was immediately used to treat the primary neurons and the other (10 µl) was imaged within four hours from the sonication using electron microscopy to confirm that the sonication was effective in generating small seeds. Forty-eight hours after treatment, cells were washed with PBS and then fixed with 4% PFA for immunofluorescence staining with anti-pS129 a-syn antibody (clone EP1536Y) (Abcam, catalog #ab51253, 1:1000).

### Proximity ligation assay (PLA)

PLA was performed on fixed primary neurons using Duolink® in situ red starter kit mouse/rabbit (Sigma–Aldrich) according to the manufacturer’s instructions. Fixed neurons were permeabilized with 0.1% Triton-X-100 in PBS for 10 min, washed three times with PBS, and incubated with blocking solution for 30 min at 37 °C in a humidity chamber. Primary antibodies (mouse anti-a-syn (clone 42) (BD Biosciences, catalog #610787, 1:500) and rabbit anti-CHMP2B (Abcam, catalog #ab33174, 1:50)) were diluted using antibody diluent. After blocking, neurons were incubated with primary antibodies at room temperature for 1 h in a humidity chamber. Coverslips were washed with wash buffer A two times for 5 min and then incubated with PLA probe solution (anti-mouse minus and anti-rabbit plus) for 1 h at 37 °C in a humidity chamber. Coverslips were washed with wash buffer A two times for 5 min. For the ligation step, 5× ligation buffer was diluted 1:5 in high purity water and ligase was added to the 1× ligation buffer at 1:40 dilution. After the wash, coverslips were incubated in the diluted ligase for 30 min at 37 °C in a humidity chamber. Post-ligation, coverslips were washed with wash buffer A two times for 5 min. For the amplification step, 5× amplification buffer was diluted 1:5 in high purity water and polymerase was added to the 1× amplification buffer at 1:80 dilution. After the wash, coverslips were incubated in the diluted polymerase for 1 h at 37 °C in a humidity chamber. Coverslips were then washed with wash buffer B two times for 10 min at room temperature with a final wash in 0.01× wash buffer B for 1 min. Coverslips were then incubated in 1× Phalloidin-iFluor 647 (Abcam) for 10 min at room temperature in the dark. Coverslips were then washed with PBS two times and mounted on slides using Duolink® in situ mounting medium with DAPI. Coverslips were allowed to dry for 15 min at room temperature and then sealed with nail polish. Slides were then imaged using a Zeiss LSM880 confocal microscope.

### Nuclear magnetic resonance (NMR)

All NMR experiments were performed on a Bruker Avance III HD 14.1 T spectrometer equipped with a cryogenically cooled, x,y,z pulse-field gradient triple-resonance probe. Resonance assignments for a-syn were confirmed by triple-resonance (HB)CBCA(CO)NNH, HNCACB, HNCO, HN(CA)CO, and HNN experiments^65^ acquired using U-{^13^C,^15^N}-labeled sample. All spectra were recorded at 25 °C.

### Surface plasmon resonance (SPR)

Kinetic interactions between peptides and CHMP2B proteins were measured at 25 °C using Biacore X-100 surface plasmon resonance (GE Healthcare). CHMP2B proteins were immobilized onto the carboxymethylated dextran surface of a CM5 sensor chip at the level of approximately 200 response units (RUs). Subsequently, a-syn monomers and oligomers, serially diluted to various concentrations (4–0.5 µM) in running buffer (50 mM Sodium acetate pH 5.5, 0.5 mM TCEP), were injected over peptides at a flow rate of 30 μl/min for 3 min with 9 min dissociation per cycle. After each cycle, surfaces were regenerated with a buffer (20 mM NaOH, 1 M NaCl, pH 10.0) for 1 min. The binding data were normalized by subtracting the response of a blank cell and then globally fitted using the BIA evaluation software to obtain kinetic interaction parameters.

### Endolysosomal flux assay

Flux via the endolysosomal pathway was measured with the Lysosome Intracellular Activity Assay Kit which was purchased from Abcam (ab234622) and used according to the manufacturer’s instructions. The kit provides a proprietary lysosome-specific self-quenched substrate which has low background fluorescence, high signal-to-background ratio, and is pH insensitive. In the assay, the substrate is taken up by cells and acts as endocytic cargo which is degraded within an endolysosomal vesicle. Following degradation, the fluorescence signal recovered from the substrate is proportional to the intracellular lysosomal activity. The activity can be measured by fluorescence microscopy or FACS. The assay was performed with HEK293T cells or *SNCA* triplication fibroblasts (obtained from NINDS Human Genetics Resource Center DNA and Cell Line Repository; NINDS Repository sample number ND27760). Cells were maintained in DMEM supplemented with 10% FBS and 1% pen/strep/glutamine. Cell medium was replaced with fresh medium containing DMSO, Leupeptin, or Bafilomycin A1 and incubated for 1 h at 37 °C with 5% CO_2_. Self-quenched substrate was added into each condition and incubated for 1 h (HEK293T cells) or overnight (*SNCA* triplication fibroblasts) at 37 °C with 5% CO_2_. After incubation, cells were washed twice in ice-cold 1× assay buffer containing DMSO, Leupeptin, or Bafilomycin A1. For HEK293T cells, cells were resuspended in PBS containing DMSO or Leupeptin and analyzed by FACS. For fibroblasts, cells were incubated in PBS containing DMSO or Bafilomycin A1 and imaged by confocal microscopy.

### Autophagic flux assay

Autophagic flux was quantitatively measured with the Autophagy LC3-HiBiT Reporter Assay System which was purchased from Promega (GA1040) and used according to the manufacturer’s instructions. When autophagy is induced, the cytosolic LC3-I fusion protein is recruited to phagophores where it becomes conjugated to phosphatidylethanolamine to form LC3-II. Upon phagophore membrane elongation and closure to form the mature autophagosome, a significant fraction of tethered LC3 protein becomes captured within the lumen together with various cargo. Subsequently, autophagosomes fuse with lysosomes to form autolysosomes, leading to the ultimate degradation of both cargo and the captured LC3 reporter protein. Thus, changes in the total level of the LC3 reporter protein can be used to monitor changes in autophagic flux. HEK293 cells (ATCC, CRL-1573) stably expressing the LC3-HiBiT reporter were trypsinized from subconfluent culture and seeded in a 10 cm plate at a density of 3.0 × 10^6^ per plate. Cells were incubated overnight at 37 °C in 5% CO_2_. Cells were transfected with FLAG-peptide plasmids in combination with pcDNA or A53T a-syn plasmids. After 24 h, cells were trypsinized and seeded in triplicate in a white flat-bottomed 96-well plate at a density of 20,000 cells per well. Cell medium was replaced the next day with fresh medium containing DMSO, Rapamycin, or Bafilomycin A1 and incubated for 24 h at 37 °C with 5% CO_2_. 80 µL of Working prewarmed Nano-Glo® HiBiT lytic buffer containing HiBiT substrate and LgBit protein (N3030) were added to each well containing 20 µL of cell medium and left to incubate on a rocker for 10 min. Samples were read using a luminometer with a 480 nm filter with measured RLU being directly proportional to HiBiT-conjugated LC3.

### *C. elegans* strains

*C. elegans* strains were grown and maintained under standard conditions at 22–23 °C ^66^. BZ555 *[dat-1p::gfp]* was obtained from the *C. elegans* Genetics Center (CGC; University of Minnesota, St Paul, MN, USA), TWH1 (*[dat-1p::a-syn(A30P), ges-1p::DsRed]; [dat-1p::gfp])* was obtained by crossing BZ555 with A30P a-syn transgenic animals (kindly provided by Dr. Takeshi Iwatsubo, University of Tokyo)^67^.

### *C. elegans* neurite length assay

The peptide construct (pPD97.78_osm-6p_TagRFP_wpeptide) was obtained by subcloning the following fragments into NheI and SpeI sites of pPD97.78 (A. Fire): NheI-AgeI *osm-1p* fragment (2.4 kb) obtained by PCR using primer set 5′-catccgctagcggatcccatggccagtggaatcacc-3′ and 5′-ccataccggtagatgtatactaatgaaggtaatagcttgaaagag-3′ and N2 genomic DNA as a template, AgeI-EcoRI *TagRFP* fragment obtained by PCR using primer set 5′-gttgaccggtATGGTGTCTAAGGGCGAAGAGCTG-3′ and 5′-ggcagaattcgaATTAAGTTTGTGCCCCAGTTTGCTAGG-3′, EcoRI-SmaI peptide (FEELEAQLARLR) fragment digested from CMV_FDDLEAQLARLR_worm, SmaI-SpeI *unc-54* 3′-UTR fragment digested from pPD95.75 (Fire vector). The TagRFP control construct was obtained by replacing the EcoRI-SmaI peptide fragment and SmaI-SpeI *unc-54* 3′-UTR fragment from pPD97.78_osm-6p_TagRFP_wpeptide, to EcoRI- SpeI *unc-54* 3′-UTR fragment digested from pPD95.75^68^.

Each plasmid (40 ng/µl) was injected with a co-injection marker, sur-5p::mCherry into TWH1. The animals carrying the extrachromosomal arrays were transferred to new plates, and their progenies were analyzed at the adult stage under a widefield microscope (Zeiss AxioObserver). Pearson Chi-square test was used for statistical analysis.

### Rats

Adult female Sprague-Dawley rats (250–280 g; Envigo) were pair-housed in cages with wood bedding and had access to food and water ad libitum. The animal colony was maintained in a regular 12 h light/dark cycle. All procedures were approved by the University Health Network Animal Care Committee in accordance with guidelines and regulations set by the Canadian Council on Animal Care (AUP #3818).

### Stereotactic surgery

Animals were secured in a stereotactic frame under isoflurane/oxygen anesthesia and ketoprofen (5 mg/kg) analgesia. The surgical site was shaved and sterilized with iodine/betadine prior to making a 2 cm incision along the midline. The scalp was exposed and a unilateral injection targeting the SN was performed at coordinates AP −5.2 mm, ML −2 mm and DV −7.5 mm with respect to the bregma as a point of reference. For each animal, a total volume of 2 μl of virus was injected at a rate of 0.5 μl/min using a microinjection pump and 10 μl Hamilton syringe with a 26-gauge needle. For A53T groups, 1 μl (low dose) or 1.34 μl (high dose) of AAV1/2-A53T a-syn (5.1 × 10^12^ genomic particles/ml), 0.14 μl of AAV1/2-PDpep1.3-GFP or Scramble1.3-GFP (5.1 × 10^12^ genomic particles/ml) and 0.86 μl or 0.52 μl of sterile PBS was injected; for EV groups, 1 μl (low dose) or 1.34 μl (high dose) of AAV1/2-EV (5.1 × 10^12^ genomic particles/ml) replaced AAV1/2-A53T. For V1S/SV2 groups, 0.58 μl of AAV1/2-V1S (1.1 × 10^12^ genomic particles/ml), 0.58 μl of AAV1/2-SV2 (1.1 × 10^12^ genomic particles/ml), 0.14 μl of AAV1/2-PDpep1.3-RFP or Scramble1.3-RFP (5.1 × 10^12^ genomic particles/ml) and 0.7 μl of sterile PBS was injected; for the YFP groups, 1.16 μl of AAV1/2-YFP (1.1 × 10^12^ genomic particles/ml), 0.14 μl of AAV1/2-PDpep1.3-RFP or Scramble1.3-RFP (5.1 × 10^12^ genomic particles/ml) and 0.7 μl of sterile PBS was injected. At the end of virus injection, the needle remained in place for 5 min before gradual removal.

### Cylinder test

Spontaneous forepaw use was evaluated using the cylinder test 1 day prior to stereotactic AAV injection, at 21 days post-injection, and at 41 days post-injection. Following overnight food restriction, individual rats with right paws marked black were placed into a glass cylinder in front of two mirrors and videos recorded. An observer blinded to treatment conditions later scored the videos by recording whether animals used their left forepaw, right forepaw, or both forepaws to touch the inner glass surface upon rearing. A total of 5 min of video recording was scored and a minimum of 10 total touches was required for data inclusion^69^.

### Brain tissue preparation

Animals were euthanised by transcardial perfusion with heparinised saline under isoflurane/oxygen anesthesia. Brains were then removed and the anterior part, including the anterior striatum, was snap frozen in liquid dry ice‐cooled isopentane. A single 1 mm thick section of the anterior striatum was immediately cut, using a matrix, for high‐performance liquid chromatography (HPLC) analysis of biogenic amines. The posterior part, including the posterior striatum, STN, and the SN, was immersion‐fixed in 4% PFA in 0.1 M PBS for 2 days and cryo‐protected in 30% sucrose in 0.1 M PBS solution for another 3 days until the brains sank. For immunofluorescent staining, 40 μm coronal cryosections were then prepared using a sliding microtome (Leica Microsystems Inc.) and 6 series of sections were stored in cryoprotectant (30% glycerol, 30% ethylene glycol, 40% PBS) at −20 °C until use.

### Catecholamine quantification by HPLC

HPLC was performed as described^47^. The investigator was blinded to experimental groups and treatment conditions. Brain sections were homogenized followed by centrifugation at 10,000 × *g* for 20 min. Catecholamines were determined from the supernatant. Values of catecholamines are expressed as ng analyte/mg total protein.

### Immunofluorescence staining of brain cryosections

Immunofluorescence staining for a-syn, pS129 a-syn, TH, and LAMP1 was performed by washing free-floating sections with PBS-T (0.1% Tween-20 or 0.2% Triton-X-100) three times for 10 min each at room temperature. Sections were then incubated in blocking solution (1% or 2% BSA, 10% normal goat serum in PBS-T) for 1 h. After blocking, sections were incubated with primary antibodies in antibody solution (2% normal goat serum in PBS-T) overnight at RT. Sections were then washed in PBS or PBS-T and incubated with secondary antibodies diluted in antibody solution for 1 h in the dark at room temperature. Sections were then mounted onto glass slides and allowed to dry and then Dako fluorescence mounting medium (Agilent) was applied, followed by coverslips.

### Human iPSC-derived dopaminergic neuron culture

A53T a-syn iCell Dopa neurons (catalog #R1109) and isogenic control iCell Dopa neurons (catalog #R1088) were purchased from Fujifilm Cellular Dynamics (Madison, WI, USA) and cultured as per the manufacturer’s instructions. Briefly, cells were plated in complete maintenance medium provided by the manufacturer on poly-L-ornithine/laminin coated μ plates (ibidi). Cells recovered from thawing for 24 h and then were transduced with AAV-Scramble1.3-RFP or AAV-PDpep1.3-RFP at MOI of 3000 for 48 h at which time media was completely changed. Cells were fixed using 4% PFA at 7 days post-plating.

### Image acquisition and analysis

Confocal images of immunofluorescent staining were acquired with a Zeiss LSM880 confocal microscope equipped with 405, 488, 555, and 639 nm laser lines. All images were taken within the linear range at constant gain and pinhole settings at optimal resolution settings determined by the software. For primary cortical neurons and iPSC-derived dopaminergic neurons, the software was programmed to acquire an image every 1 μm for a total of 11 μm, capturing all of the neurons visible in the z-plane in each field of view using a ×63 objective. At least 30 images were acquired for each treatment group from three independent experiments. For animal experiments, the whole midbrain or striatum regions were imaged using a ×5 or ×10 objective. Ten serial coronal midbrain sections were imaged per animal, separated by 240 μm intervals. One to six images of the striatum per animal were acquired and a representative image of a single coronal section present in all sets was chosen for analysis, based on anatomical features.

Confocal images of immunofluorescent staining of midbrain sections were processed using HALO software (Indica Labs), which is a well validated tool for automatic quantification of neurons in brain tissue sections^70–72^. Initially, ipsilateral SN was selected as a region of interest (ROI). Dopaminergic neurons were subsequently identified by automated detection of TH-labeled objects within this ROI, as previously validated by correlation analyses with traditional stereological methods^48,73^. Levels of total a-syn were assessed in this ROI by measuring the area and intensity of anti-a-syn staining. Confocal images of immunofluorescent staining of striatal sections were processed using HALO software (Indica Labs) or ImageJ software (Fiji). Ipsilateral striatum was initially selected as a ROI based on density of TH-labeled dopaminergic terminals within this ROI. Fluorescent intensity and area were then assessed in this ROI.

Confocal images of primary cortical neurons, iPSC-derived dopaminergic neurons, and LAMP1 staining within the SN were processed using Imaris software (Oxford instruments). Z stacks were projected to give a 3D reconstruction of the field of view. Mean pixel intensity per GFP^+^ cell (for experiments using GFP-tagged peptides) or RFP^+^ cell (for experiments using RFP-tagged peptides) was calculated for a-syn or LAMP1 signal using the software’s surfaces module. The software’s surfaces module was used to identify GFP^+^/NeuN^+^ cells, and number of pS129 a-syn^+^ puncta per GFP^+^/NeuN^+^ cortical neuron was counted using the software’s spots module. Mean pixel intensity per RFP^+^/NeuN^+^ cortical neuron was calculated for Rab7, cathepsin B, or cathepsin D signals using the software’s surfaces module. For LAMP1 staining within the SN, the number of LAMP1^+^ puncta within GFP^+^ cells was calculated for each field of view; 3 fields of view were imaged per animal.

### Statistical analysis

All data are represented as mean ± s.d. with at least three independent experiments, unless otherwise stated. Statistical analysis was performed using GraphPad Prism 8 or SPSS. For *t*-tests, Student’s *t*-tests were performed unless otherwise stated.

### Reporting summary

Further information on research design is available in the Nature Portfolio Reporting Summary linked to this article.

## Supplementary information

Supplementary Information

Reporting Summary

## Data Availability

All relevant data supporting the key findings of this study are available within the main manuscript and its Supplementary Information. Source data are provided with this paper.

## Source data

Source Data
