# Supplementary material for: Disrupting the α-synuclein-ESCRT interaction with a peptide inhibitor mitigates neurodegeneration in preclinical models of Parkinson’s disease
Source: Nat Commun. 2023 Apr 19;14:2150. doi: 10.1038/s41467-023-37464-2 (PMC10115881; doi:10.1038/s41467-023-37464-2)
Supplement: Supplementary file 1 — Supplementary Information [file 41467_2023_37464_MOESM1_ESM.pdf]

Supplementary Information for

Disrupting the α-synuclein-ESCRT interaction with a peptide inhibitor mitigates neurodegeneration in preclinical models of Parkinson's disease

Satra Nim[#], Darren M. O'Hara[#], Carles Corbi-Verge, Albert Perez-Riba, Kazuko Fujisawa, Minesh Kapadia, Hien Chau, Federica Albanese, Grishma Pawar, Mitchell L. De Snoo, Sophie G. Ngana, Jisun Kim, Omar M. A. El-Agnaf, Enrico Renella, Lewis E. Kay, Suneil K. Kalia[#], Lorraine V. Kalia[#], Philip M. Kim[#]

[#]These authors contributed equally

This PDF file includes:

Figures S1 to S6
Tables S1

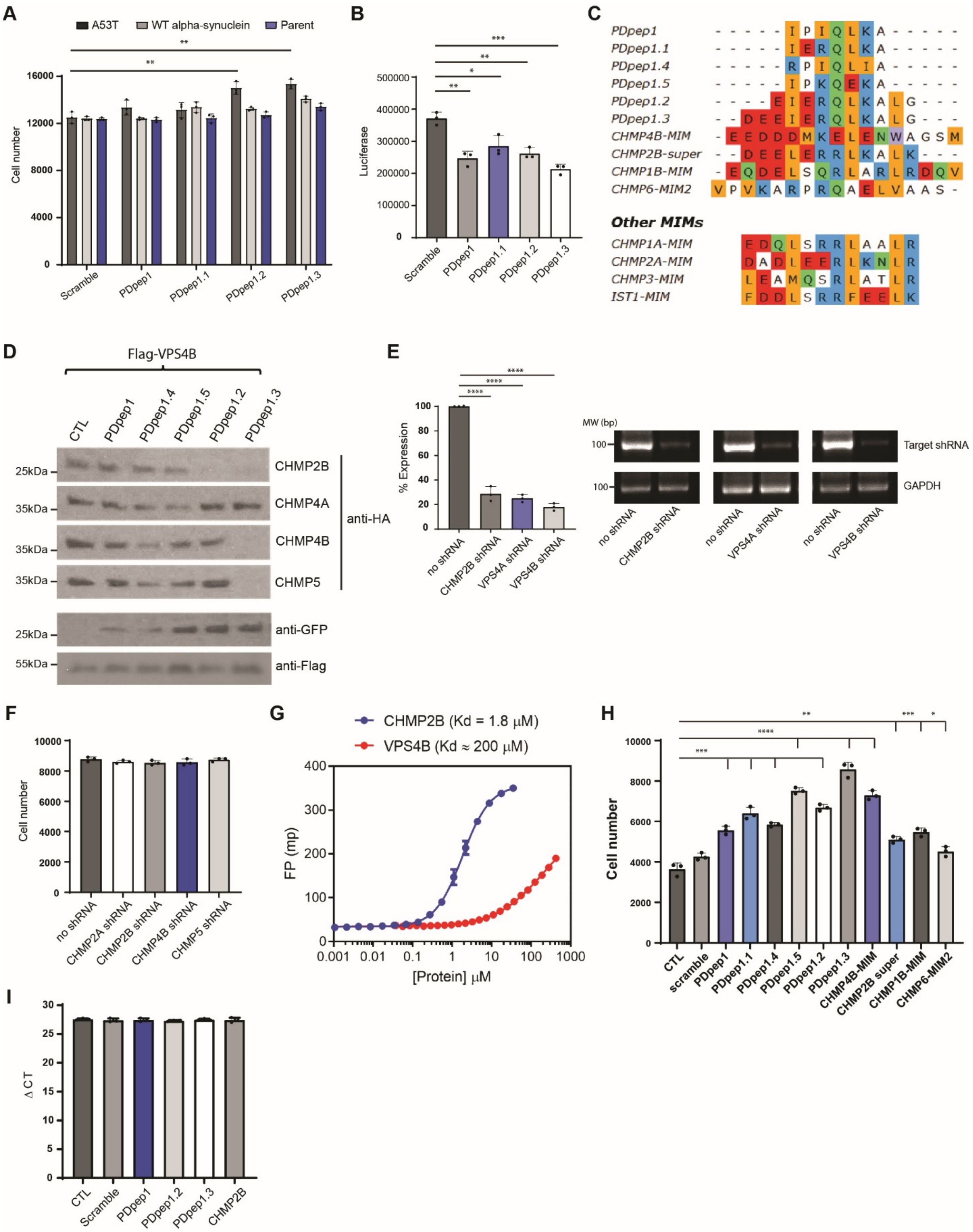
A
A53T
WT alpha-synuclein
Parent
Cell number
Scramble
PDpep1
PDpep1.1
PDpep1.2
PDpep1.3
B
Luciferase
C
PDpep1
PDpep1.1
PDpep1.4
PDpep1.5
PDpep1.2
PDpep1.3
CHMP4B-MIM
CHMP2B-super
CHMP1B-MIM
CHMP6-MIM2
Other MIMs
CHMP1A-MIM
CHMP2A-MIM
CHMP3-MIM
IST1-MIM
D
Flag-VPS4B
CTL
PDpep1
PDpep1.4
PDpep1.5
PDpep1.2
PDpep1.3
CHMP2B
CHMP4A
CHMP4B
CHMP5
anti-HA
anti-GFP
anti-Flag
E
% Expression
no shRNA
CHMP2B shRNA
VPS4A shRNA
VPS4B shRNA
MW (bp)
Target shRNA
GAPDH
F
Cell number
no shRNA
CHMP2A shRNA
CHMP2B shRNA
CHMP4B shRNA
CHMP5 shRNA
G
CHMP2B (Kd = 1.8 μM)
VPS4B (Kd ≈ 200 μM)
FP (mp)
[Protein] μM
H
Cell number
CTL
scramble
PDpep1
PDpep1.1
PDpep1.4
PDpep1.5
PDpep1.2
PDpep1.3
CHMP4B-MIM
CHMP2B super
CHMP1B-MIM
CHMP6-MIM2
I
ΔCT
CTL
Scramble
PDpep1
PDpep1.2
PDpep1.3
CHMP2B

Figure S1. Additional *in vitro* validation of hits, PDpep1.3 disrupts CHMP2B-VPS4B interaction and binds more tightly to CHMP2B than VPS4B. (A) Validation of cell viability effect of peptides in A53T or (wild type (WT) a-syn expressing cells without addition of MG132. Data represent means ± s.d. (unpaired two-tailed t-tests; PDpep1.2, *P*=0.0040; PDpep1.3, *P*=0.0016; n=3). **(B)** A53T a-syn oligomers as measured by luciferase activity. All 4 peptides showed significant reduction in A53T a-syn oligomers in cells stably expressing split luciferase a-syn constructs. Data represent means ± s.d. (unpaired two-tailed t-tests; PDpep1, *P*=0.0019; PDpep1.1, *P*=0.0166; PDpep1.2, *P*=0.0020; PDpep1.3, *P*=0.0004; n=3). **(C)** Overlapping sequences of different MIMs as displayed in Ugene (*1*) and aligned with ClustalW (*2*). CHMP1B-MIM represents a classic MIM, and CHMP2B-super is a version of PDpep1.3 modified to be closer to a classic MIM. **(D)** Co-immunoprecipitation experiments to test for PPI disruption. Flag-VPS4B was immunoprecipitated in the presence of different HA-tagged CHMP proteins and GFP-tagged peptides. PDpep1.3 disrupted the interaction between VPS4B and CHMP2B. We used GFP alone as a control (CTL). **(E)** Reduction in gene expression level with shRNA knockdown. Expression of targeted gene was measured in cells stably expressing shRNA as indicated. Experiments were done in triplicate. Data represent means ± s.d. (unpaired two-tailed t-tests; CHMP2B shRNA, *P*=<0.0001; VPS4A shRNA, *P*<0.0001; VPS4B shRNA, *P*<0.0001; n=3). **(F)** Effects of shRNA gene knockdown in A53T a-syn expressing cells on cell viability. Data represent means ± s.d. (n=3). **(G)** Fluorescence polarization (FP) binding assay of a FITC-labeled DEEIERQLKALG peptide against CHMP2B (*Kd*=1.8 µM) (blue) and VPS4B (*Kd* ~200 µM) (red). **(H)** Effect of MIM motif peptides (illustrated in **C**) on cell viability in A53T a-syn expressing cells. We used GFP alone as a control (CTL). Data represent mean values ± s.d. (unpaired two-tailed t-tests; PDpep1, *P*=0.0008; PDpep1.1, *P*=0.0004; PDpep1.4, *P*=0.0003; PDpep1.5, *P*<0.0001; PDpep1.2, *P*=0.0001; PDpep1.3, *P*<0.0001; CHMP4B-MIM, *P*<0.0001; CHMP2B-super, *P*=0.0019; CHMP1B-MIM, *P*=0.0010; CHMP6-MIM2, *P*=0.0181; n=3). **(I)** ΔCt values for the RT-PCR data in Figure 2A showing no difference in a-syn levels. Data represent means ± s.d. (n=3). **P*<0.05; ***P*<0.01; ****P*<0.001; *****P*<0.0001. Source data are provided as a Source Data file.

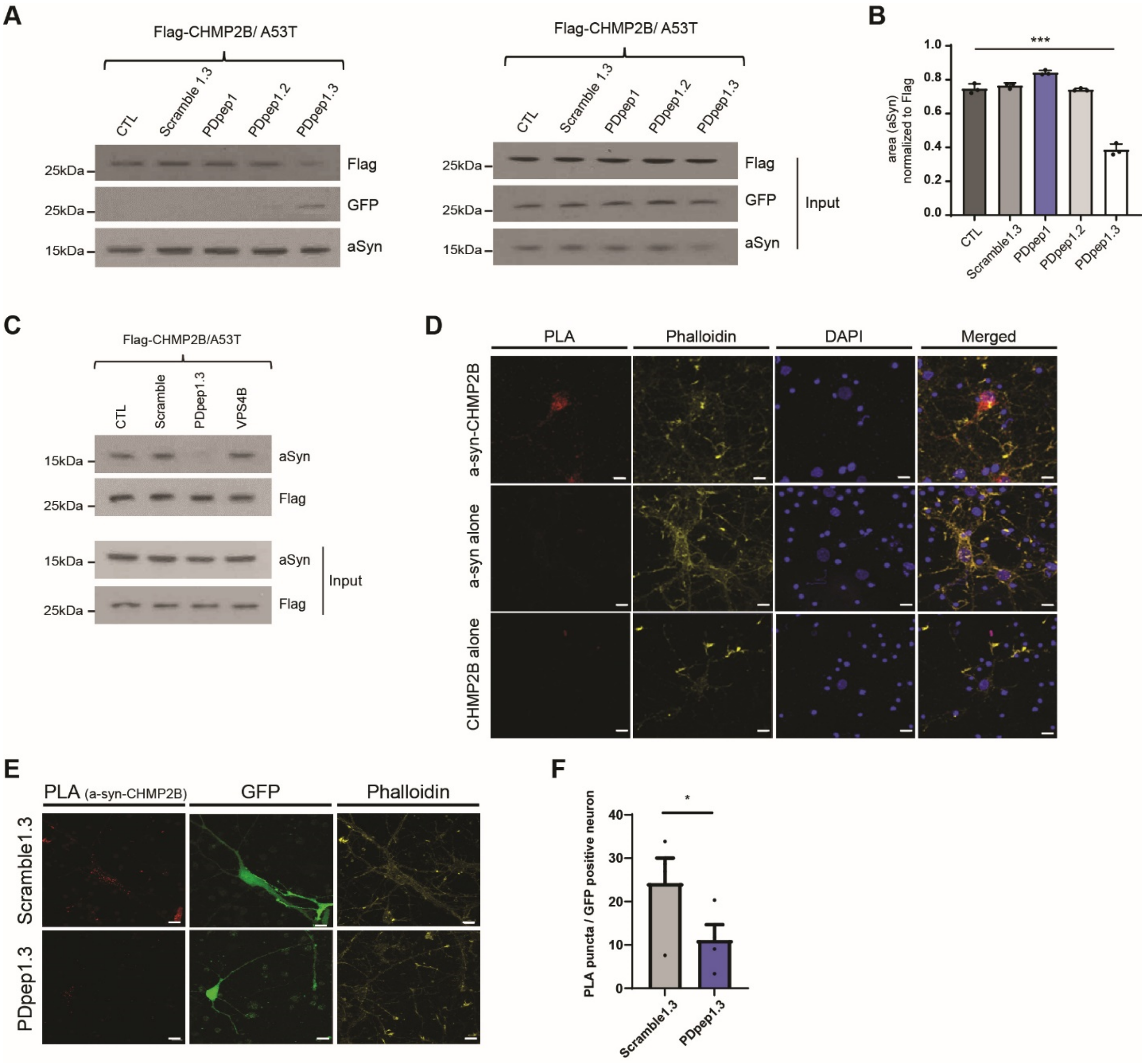

Figure S2. PDpep1.3 outcompetes a-syn binding to CHMP2B. (A) Validation of PPI disruption using reverse co-immunoprecipitation assays. HA-tagged A53T a-syn was immunoprecipitated in the presence of Flag-CHMP2B and GFP-tagged peptides. For controls, we included GFP alone (CTL) and a GFP-tagged scrambled version PDpep1.3 (Scramble1.3). **(B)** Quantification of immunoblot bands for a-syn (normalized to Flag) from Figure 3A (left panel). Data represent means ± s.d. (unpaired two-tailed t-test, $t(4)=2.7764$; PDpep1.3, $P=0.0002$; n=3). **(C)** Validation of PPI disruption using co-immunoprecipitation assays. Flag-CHMP2B was immunoprecipitated in the presence of HA-tagged A53T a-syn and GFP-tagged peptides or VPS4B (n=3). **(D)** Representative confocal images of *in situ* proximity ligation assay (PLA) signal (red) indicating a physical interaction between a-syn and CHMP2B in rat primary cortical neurons (stained with phalloidin) (top panels). Negative controls for the assay include anti-a-syn antibodies alone (middle panels) or anti-CHMP2B antibodies alone (bottom panels) (n=10 fields of view; scale bars=5 μm). **(E)** Representative confocal images of *in situ* PLA signal, indicating an a-syn-CHMP2B interaction, which is lower in rat primary cortical neurons transduced with PDpep1.3-

GFP compared with Scramble1.3-GFP (scale bars=5 μm). **(F)** Quantification of number of PLA puncta in GFP positive neurons. Bars represent means ± s.e.m. (unpaired two-tailed t-test, $t(18)= 2.881$; $P=0.0099$; n=4). **$P<0.01$; ***$P<0.001$. Source data are provided as a Source Data file.

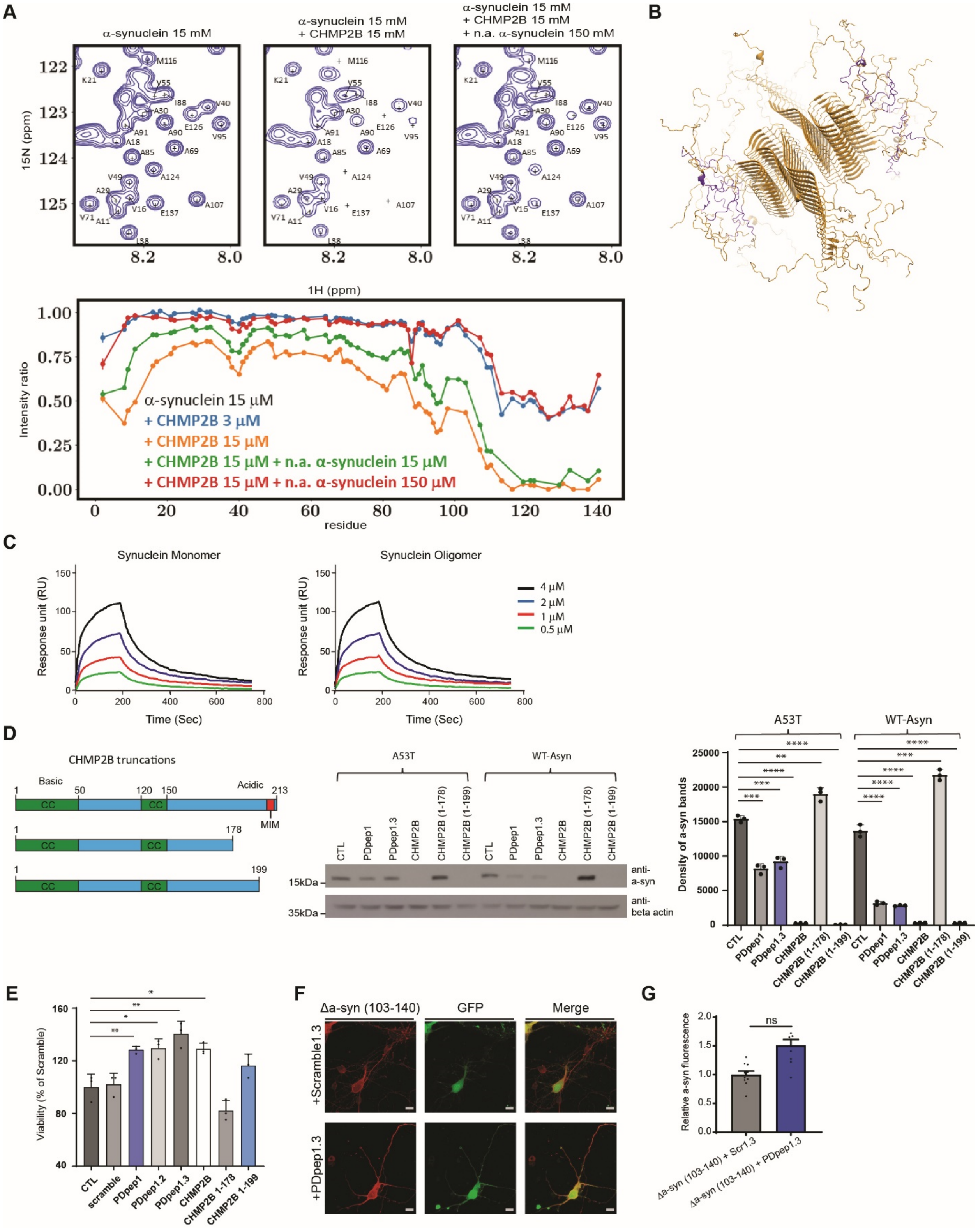

Figure S3. CHMP2B interacts with the C-terminus of a-syn. (A) Interaction between a-syn and CHMP2B probed by solution NMR spectroscopy. Top: Regions of 15N,1H HSQC spectra for U-15N a-syn alone (left panel), in the presence of 1:1 CHMP2B (middle panel), and in the presence

of both 1:1 CHMP2B and 10-fold excess of natural abundance (n.a.) a-syn (right panel). Bottom: Ratios of intensities vs. residue number. The numerator of the ratios is indicated by the figure legend, while the denominator is the intensity of U-15N a-syn alone. The profiles for the samples in red and blue indicate that approximatively 12 μM of CHMP2B are sequestered by the presence of 150 μM a-syn and corresponding to a *Kd* of ~20 μM. **(B)** Structure of a-syn and binding region to CHMP2B (in violet). PDB ID is 2N0A, and the residues in violet are 103-NEEGAPQEGILE-114. **(C)** Surface plasmon resonance (SPR) binding assay of CHMP2B against a-syn monomer (*Kd*=1.82 μM) and a-syn oligomer (*Kd*=1.37 μM). **(D)** Schematic of CHMP2B truncations. PDpep1, PDpep1.3, and CHMP2B 1-199 truncation are each associated with a reduction in a-syn levels by immunoblot. Quantification of a-syn levels is shown in the bar graph. Data represent means ± s.d. (unpaired two-tailed t-tests; A53T PDpep1, P=0.0001; A53T PDpep1.3, P=0.0004; A53T CHMP2B, P<0.0001; A53T CHMP2B (1-178), P=0.0044; A53T CHMP2B (1-199), P<0.0001; WT-Asyn PDpep1, P<0.0001; WT-Asyn PDpep1.3, P=0.0001; WT-Asyn CHMP2B, P<0.0001; WT-Asyn CHMP2B (1-178), P=0.0003; WT-Asyn CHMP2B (1-199), P<0.0001; n=3). **(E)** Cell viability assay in A53T a-syn cell line with peptides and truncated CHMP2B proteins. Data represent means ± s.d. (unpaired two-tailed t-tests; PDpep1, P=0.0091; PDpep1.2, P=0.0143; PDpep1.3, P=0.0073; CHMP2B, P=0.0101; n=3). **(F)** Representative images of rat primary cortical neurons transduced with a truncated version of A53T a-syn lacking amino acids 103-140 (Δa-syn (103-140)) which was HA-tagged plus Scramble1.3-GFP or PDpep1.3-GFP (scale bars=5 μm). **(G)** Quantification of relative Δa-syn(103-140) fluorescence (by immunostaining with anti-HA antibodies) in GFP positive neurons. Bars represent means ± s.e.m. (two-tailed nested t-test, $t(57)$=2.722; P=0.0529; n=3). *P<0.05; **P<0.01; ***P<0.001; ****P<0.0001; ns indicates P>0.05. Source data are provided as a Source Data file.

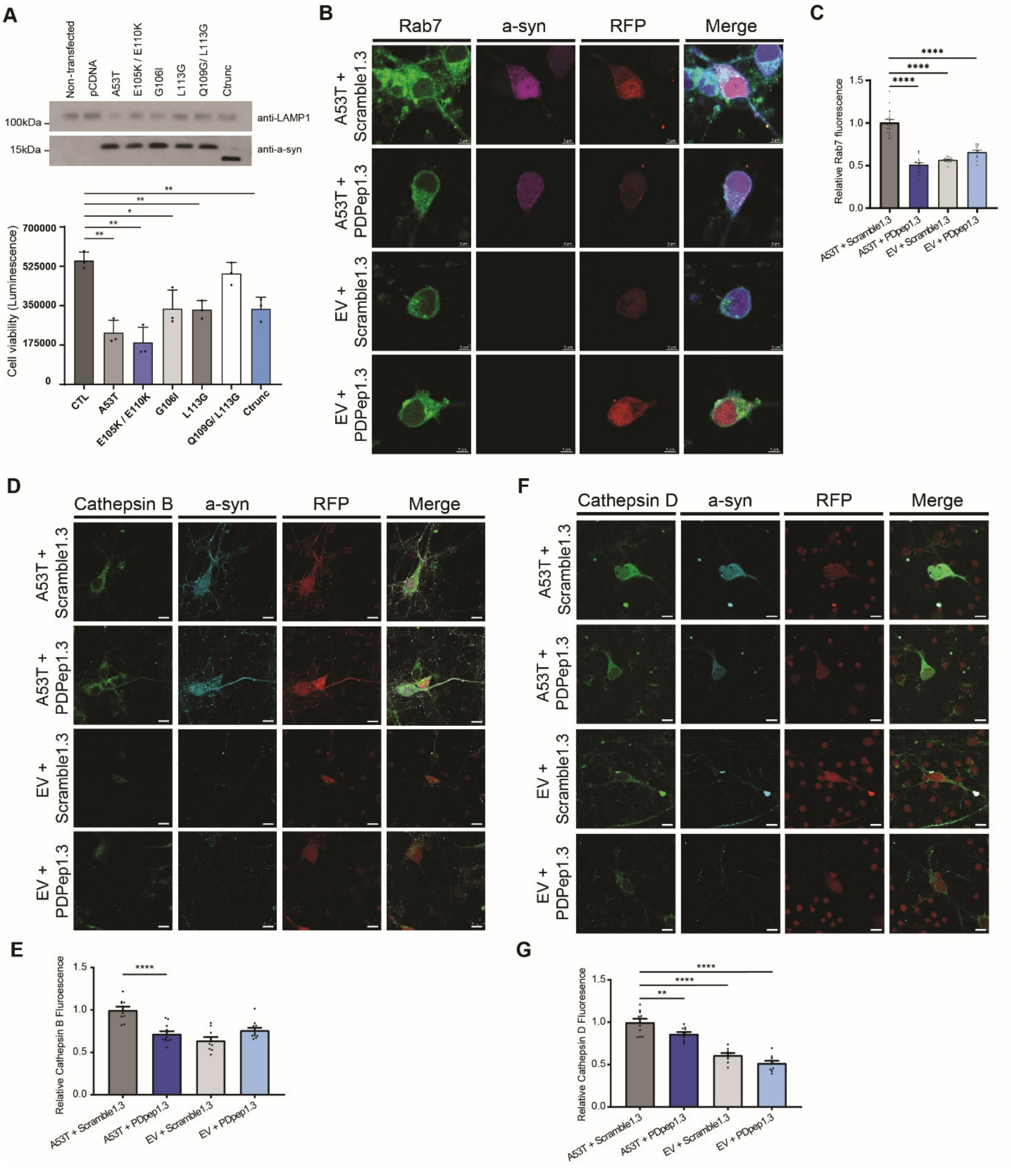

Figure S4. Endolysosomal markers are disrupted by a-syn and normalized by PDpep1.3. (A) Effects of a-syn mutations in the CHMP2B binding region on LAMP1 levels (upper panel) and cell viability (lower panel). Data represent means ± s.d. (unpaired two-tailed t-tests; A53T, *P*=0.0012; E105K/E110K, *P*=0.0013; G106I, *P*=0.0154; L113G, *P*=0.0024; Ctrunc, *P*=0.0044; n=3). **(B)** Representative images of primary cortical neurons transduced with A53T a-syn or EV plus Scramble1.3-RFP or PDpep1.3-RFP and immunostained for Rab7 and a-syn (scale bars=5

μm). **(C)** Quantification of Rab7 fluorescence in RFP positive neurons. Bars represent means ± s.e.m. (one-way ANOVA, $F(3, 142)=43.07$; $P<0.0001$ followed by Dunnett's post-test; n=3). **(D)** Representative images of rat primary cortical neurons transduced with A53T a-syn or EV plus Scramble1.3-RFP or PDpep1.3-RFP and immunostained for cathepsin B and a-syn (scale bars=10 μm). **(E)** Quantification of cathepsin B fluorescence in RFP positive neurons. Bars represent means ± s.e.m. (one-way ANOVA, $F(3, 40)=18.44$; $P<0.0001$ followed by Dunnett's post-test; n=3). **(F)** Representative images of rat primary cortical neurons transduced with mutant A53T a-syn or EV plus Scramble1.3-RFP or PDpep1.3-RFP and immunostained for cathepsin D and a-syn (scale bars=10 μm). **(G)** Quantification of cathepsin D fluorescence in RFP positive neurons. Bars represent means ± s.e.m. (one-way ANOVA, $F(3, 40)=57.07$; $P<0.0001$ followed by Dunnett's post-test; n=3). *$P<0.05$; **$P<0.01$; ****$P<0.0001$. Source data are provided as a Source Data file.

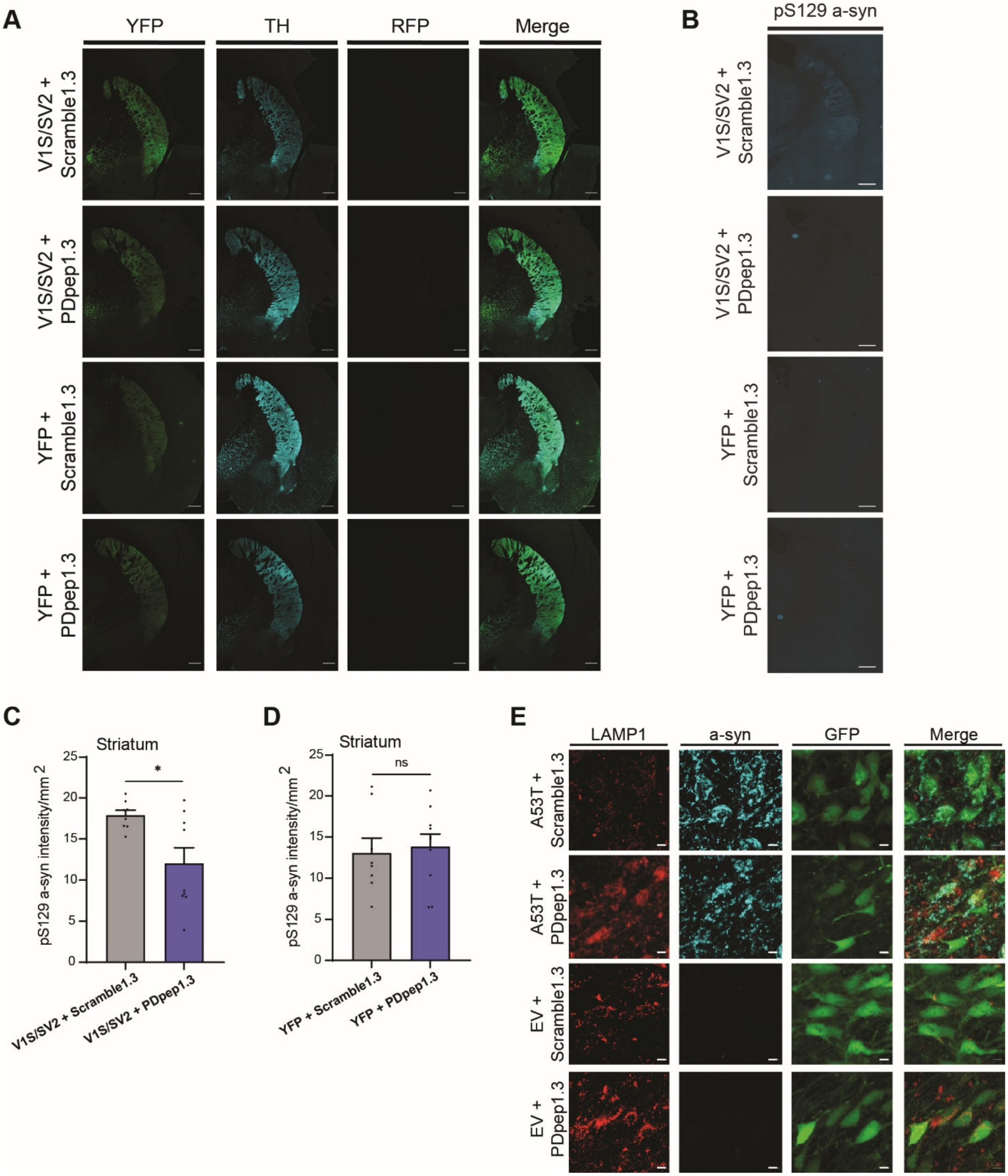

Figure S5. PDpep1.3 rescues TH and pS129 a-syn levels in striatum and increases LAMP1 levels in SN. (A) Representative images of the striatum of rats injected with V1S/SV2 or YFP plus Scramble1.3-RFP or PDpep1.3-RFP showing **(A)** immunostaining with anti-tyrosine hydroxylase (TH) antibody and native fluorescence (YFP or RFP) or **(B)** immunostaining with anti-pS129 a-syn antibody (scale bars=500 µm). Quantification of pS129 a-syn fluorescence in rat striatum at 6 weeks post injection of **(C)** V1S/SV2 plus Scramble1.3-RFP or PDpep1.3-RFP (unpaired two-

tailed t-test, $t(15)=2.787$; $P=0.0138$; Scramble1.3, n=8 rats; PDpep1.3, n=9 rats) or **(D)** full length YFP plus Scramble1.3-RFP or PDpep1.3-RFP (unpaired two-tailed t-test, $t(16)=1.040$; $P=0.31$; Scramble1.3, n=8 rats; PDpep1.3, n=10 rats). Bars represent means ± s.e.m. **(E)** Representative z-stack projection images showing immunostaining (with anti-LAMP1 or anti-a-syn antibodies) and native GFP fluorescence in substantia nigra (SN) of rats injected with low-titer A53T a-syn plus Scramble-GFP or PDpep1.3-GFP (top two panels); or low-titer EV plus Scramble-GFP or PDpep1.3-GFP (bottom two panels) (scale bars=10 µm). *$P<0.05$; ns indicates $P>0.05$. Source data are provided as a Source Data file.

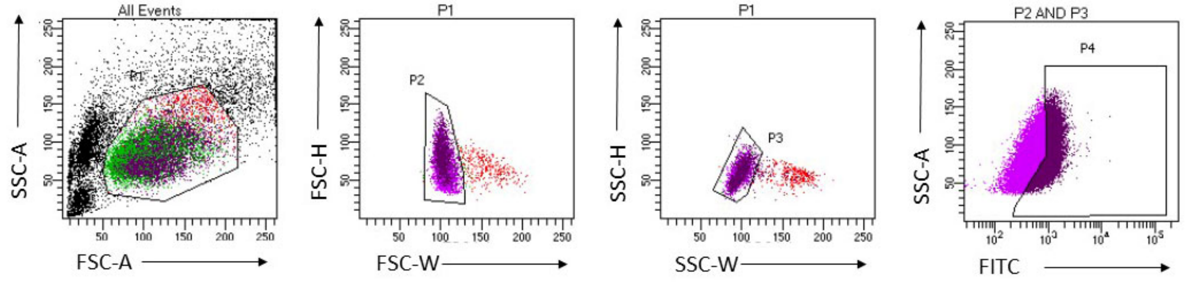

Figure S6. Flow cytometry gating. Representative flow cytometric gating hierarchy used for data shown in Figure 3F.

WT a-syn cytotoxicity screen							
Peptide	Reads 0µM MG132	Reads 10µM MG132	Reads 25µM MG132	Reads 50µM MG132	Slope Norm Reads	# Mapped Complexes	# PD GO Terms
**IPIQLKA**	90	1	1629	3375	3.45	11	16
RAPSCHL	1	13	33	-	0.15	2	50
DWLMVNL	1	13	29	-	0.13	3	5
LPASMPC	2	16	27	-	0.09	1	0
LQVGVAV	1	25	23	-	0.09	13	18
PLPTGMG	1	3	20	-	0.08	7	8
CMVHSAG	4	14	33	-	0.08	1	1
AAYNLCA	1	14	19	-	0.07	6	18
LSGECVP	3	28	28	-	0.07	9	27
GAAAGGT	1	10	17	-	0.06	70	162

A53T a-syn cytotoxicity screen							
Peptide	Reads 0µM MG132	Reads 10µM MG132	Reads 25µM MG132	Reads 50µM MG132	Slope Norm Reads	# Mapped Complexes	# PD GO Terms
**IPIQLKA**	168	20	3859	13308	40.54	11	16
QSVVTAP	8	6	37	465	1.38	16	30
CKYRSVL	5	7	11	438	1.30	4	9
PAMQIDG	32	20	53	337	0.96	6	4
GWTTVRR	15	1	29	322	0.94	0	0
QSVLQQV	13	2	12	282	0.82	8	37
QSPSSAW	9	45	59	284	0.82	19	46
QVSVAQQ	45	13	120	246	0.69	9	18
QAELSNS	6	8	10	235	0.69	5	39
EDVNKCV	6	6	13	233	0.68	4	11

A53T a-syn oligomers screen						
Peptide	Reads GFP Neg	Reads GFP Inter	Reads GFP High	Reads Ratio	# Mapped Complexes	# PD GO Terms
**IPIQLKA**	15556	2853	12	1,218.98	11	16
WPRYPHI	92	0	1	47.36	2	1
CFLFQIQ	88	0	1	45.32	0	0
ECHTKIR	76	0	1	39.21	2	9
MQMKLSQ	70	0	1	36.15	6	45
TQKRELT	69	0	1	35.64	7	21
VYLDILG	59	0	1	30.55	7	2
LKRDQPV	118	0	3	30.30	21	103
NNQYSFV	58	0	1	30.04	10	29
LPSKIYK	57	0	1	29.53	14	167

Table S1. Peptide counts from the screens. Illumina sequencing results after demultiplexing, demonstrating peptide counts. Only reads with an average quality Phred score of >30 were

selected, and frequencies were calculated. Reads were then normalized to the total number of peptides read for that sample population, and a scalar factor was applied.

Supplementary References:

1. K. Okonechnikov, O. Golosova, M. Fursov, Unipro UGENE: a unified bioinformatics toolkit. *Bioinformatics* **28**, 1166-1167 (2012).
2. M. A. Larkin *et al.*, Clustal W and Clustal X version 2.0. *Bioinformatics* **23**, 2947-2948 (2007).
