# Supplementary material for: Disrupting the α-synuclein-ESCRT interaction with a peptide inhibitor mitigates neurodegeneration in preclinical models of Parkinson’s disease
Source: Nat Commun. 2023 Apr 19;14:2150. doi: 10.1038/s41467-023-37464-2 (PMC10115881; doi:10.1038/s41467-023-37464-2)
Supplement: Supplementary file 2 — Reporting Summary [file 41467_2023_37464_MOESM2_ESM.pdf]

nature portfolio

Corresponding author(s): Suneil K. Kalia, Lorraine V. Kalia, Philip M. Kim

Last updated by author(s): Feb 18, 2023

Reporting Summary

Nature Portfolio wishes to improve the reproducibility of the work that we publish. This form provides structure for consistency and transparency in reporting. For further information on Nature Portfolio policies, see our Editorial Policies and the Editorial Policy Checklist.

Statistics

For all statistical analyses, confirm that the following items are present in the figure legend, table legend, main text, or Methods section.

n/a	Confirmed	
☐	☒	The exact sample size (*n*) for each experimental group/condition, given as a discrete number and unit of measurement
☐	☒	A statement on whether measurements were taken from distinct samples or whether the same sample was measured repeatedly
☐	☒	The statistical test(s) used AND whether they are one- or two-sided<br>*Only common tests should be described solely by name; describe more complex techniques in the Methods section.*
☒	☐	A description of all covariates tested
☐	☒	A description of any assumptions or corrections, such as tests of normality and adjustment for multiple comparisons
☐	☒	A full description of the statistical parameters including central tendency (e.g. means) or other basic estimates (e.g. regression coefficient) AND variation (e.g. standard deviation) or associated estimates of uncertainty (e.g. confidence intervals)
☐	☒	For null hypothesis testing, the test statistic (e.g. F, t, r) with confidence intervals, effect sizes, degrees of freedom and P value noted<br>*Give P values as exact values whenever suitable.*
☒	☐	For Bayesian analysis, information on the choice of priors and Markov chain Monte Carlo settings
☒	☐	For hierarchical and complex designs, identification of the appropriate level for tests and full reporting of outcomes
☒	☐	Estimates of effect sizes (e.g. Cohen's *d*, Pearson's *r*), indicating how they were calculated

Our web collection on statistics for biologists contains articles on many of the points above.

Software and code

Policy information about availability of computer code

Data collection	Imaris ver 9 (Oxford instruments) was used for analysis of confocal images of cultured cells. HALO (Indica labs) or ImageJ software (Fiji) was used for image analysis of immunostained brain sections from in vivo studies.
Data analysis	Statistical analyses were performed using GraphPad Prism 8 or SPSS. The enrichment term analysis was performed using DAVID based on the list of 330 genes annotated by the Parkinson's Disease Gene Ontology Annotation Institute at University College London.

For manuscripts utilizing custom algorithms or software that are central to the research but not yet described in published literature, software must be made available to editors and reviewers. We strongly encourage code deposition in a community repository (e.g. GitHub). See the Nature Portfolio guidelines for submitting code & software for further information.

Data

Policy information about availability of data

All manuscripts must include a data availability statement. This statement should provide the following information, where applicable:

- Accession codes, unique identifiers, or web links for publicly available datasets
- A description of any restrictions on data availability
- For clinical datasets or third party data, please ensure that the statement adheres to our policy

The data that support the findings of this study are available, and a complete data availability statement in the manuscript under a separate "Data Availability" section is provided.

Human research participants

Policy information about studies involving human research participants and Sex and Gender in Research.

Reporting on sex and gender	N/A
Population characteristics	N/A
Recruitment	N/A
Ethics oversight	N/A

Note that full information on the approval of the study protocol must also be provided in the manuscript.

Field-specific reporting

Please select the one below that is the best fit for your research. If you are not sure, read the appropriate sections before making your selection.

☒ Life sciences ☐ Behavioural & social sciences ☐ Ecological, evolutionary & environmental sciences

For a reference copy of the document with all sections, see nature.com/documents/nr-reporting-summary-flat.pdf

Life sciences study design

All studies must disclose on these points even when the disclosure is negative.

Sample size	For in vitro experiments, a standard sample size of at least 3 was used to ensure data could be reproduced. We determined this to be sufficient as we included all necessary controls and observed low variability between samples. For in vivo experiments, previous work from our lab and others demonstrated that a sample size of at least 5 animals per group is sufficient to detect statistically significant changes in dopaminergic neuron density in the substantia nigra (Chen et al PMID 34772429; McKinnon et al PMID 32059750).
Data exclusions	Rats in which stereotactic injection was mistargeted were excluded from all analyses. These animals were identified by counter-staining for TH in brain sections; animals in which there was no overlap between GFP/RFP-PDpep1.3/RFP-Scr1.3 signal and TH signal were deemed as mistargeted. Rats which did not touch the cylinder wall a minimum of 10 times in 5 minutes of recording were excluded from behavioural analysis only (as detailed in the Method section).
Replication	For in vitro experiments, each experiment was repeated at least 3 times and statistical analysis was carried out using GraphPad Prism to identify any significant differences between groups. All attempts at replication were successful. For in vivo experiments, all animals were treated in the same manner and previous work from our lab and others has shown the samples sizes chosen are sufficient to determine significant differences.
Randomization	To ensure that average body weight did not differ between experimental groups, rats were weighed prior to stereotactic injection and sorted in descending order based on body weight. They were then assigned to each group (e.g., groups A to D) in order (i.e., the heaviest animal was assigned to group A (A53T/PDpep1.3), 2nd heaviest to group B (A53T/Scr1.3), 3rd heaviest to group C (EV/PDpep1.3), 4th heaviest to group D (EV/Scr1.3), 5th heaviest to group A (A53T/PDpep1.3), 6th heaviest to group B (A53T/Scr1.3), and so on).
Blinding	After sorting into groups, animals were randomly assigned numbers and experimenters remained blinded to the groups until all data analyses were completed. For other experiments, data collection and/or analysis were not blinded as analysis was not impacted by this (e.g., intensity on Western Blots or software analysis with identical image acquisition settings).

Reporting for specific materials, systems and methods

We require information from authors about some types of materials, experimental systems and methods used in many studies. Here, indicate whether each material, system or method listed is relevant to your study. If you are not sure if a list item applies to your research, read the appropriate section before selecting a response.

Materials & experimental systems

n/a	Involved in the study
☐	☒ Antibodies
☐	☒ Eukaryotic cell lines
☒	☐ Palaeontology and archaeology
☐	☒ Animals and other organisms
☒	☐ Clinical data
☒	☐ Dual use research of concern

Methods

n/a	Involved in the study
☒	☐ ChIP-seq
☐	☒ Flow cytometry
☒	☐ MRI-based neuroimaging

Antibodies

Antibodies used

Figure 1D
Primary antibodies
o Anti-GFP (1:2000; Rabbit polyclonal, Abcam, ab290)
o In-lab Validation: Antibody tested in non-transduced or non-transfected negative control HEK293 cells.
o Manufacturer Validation: Antibody specificity was demonstrated by detecting the GFP fraction from cell extracts expressing recombinant GFP fusion proteins and has also been shown to be useful on mouse sections fixed with formalin. https://www.abcam.com/gfp-antibody-ab290.html
o Anti-Flag HRP (1:1000; Mouse monoclonal, Sigma, A8592)
o In-lab Validation: Antibody tested in non-transduced or non-transfected negative control HEK293 cells.
o Manufacturer Validation: Specificity routinely tested by western blot analysis. https://www.sigmaaldrich.com/CA/en/product/sigma/a8592
Secondary antibodies
o Anti-rabbit HRP-linked (1:5000; Cell Signaling, 7074)
o Manufacturer Validation: This product is thoroughly validated with CST primary antibodies and will work optimally with the CST western immunoblotting protocol, ensuring accurate and reproducible results. https://www.cellsignal.com/products/secondary-antibodies/anti-rabbit-igg-hrp-linked-antibody/7074

Figure 2A
Primary antibodies
o Anti-a-syn (1:1000; Mouse monoclonal, clone 42, BD Biosciences, 610787)
o In-lab Validation: Specificity tested by Western blot in transfected cells.
o Manufacturer Validation: Specificity routinely tested by western blot analysis. https://www.bdbiosciences.com/en-ca/products/reagents/microscopy-imaging-reagents/immunofluorescence-reagents/purified-mouse-anti-synuclein.610787
o Anti β-Actin (1:1000; Rabbit monoclonal, clone 13E5, Cell Signaling Technologies, 4970)
o Manufacturer Validation: Antibody specificity was demonstrated by western blot analysis of extracts from various cell types as well as western blot analysis of recombinant Actin isoforms using a Pan-Actin Antibody #4968. https://www.cellsignal.com/products/primary-antibodies/b-actin-13e5-rabbit-mab/4970
Secondary antibodies
o Anti-mouse HRP-linked (1:5000; Cell Signaling, 7076)
o In-lab Validation: No primary (secondary only) negative controls.
o Manufacturer Validation: This product is thoroughly validated with CST primary antibodies and will work optimally with the CST western immunoblotting protocol, ensuring accurate and reproducible results. https://www.cellsignal.com/products/secondary-antibodies/anti-mouse-igg-hrp-linked-antibody/7076
o Anti-rabbit HRP-linked (1:5000; Cell Signaling, 7074)
o Manufacturer Validation: This product is thoroughly validated with CST primary antibodies and will work optimally with the CST western immunoblotting protocol, ensuring accurate and reproducible results. https://www.cellsignal.com/products/secondary-antibodies/anti-rabbit-igg-hrp-linked-antibody/7074

Figure 2D
Primary antibodies
o Anti-a-syn (1:500; Mouse monoclonal, clone SYN 211, Invitrogen, 32-8100)
o In-lab Validation: Antibody tested in rat primary cortical neurons and rat brain tissue as described in the methods section.
o Manufacturer Validation: Antibody specificity was demonstrated by CRISPR-Cas9 mediated knockout of target protein. A loss of signal was observed for target protein in SNCA (KO) cell line compared to control cell line using Anti-SNCA Polyclonal Antibody (Product # 32-8100). https://www.thermofisher.com/antibody/product/alpha-Synuclein-Antibody-clone-Syn-211-Monoclonal/32-8100
Secondary antibodies
o AlexaFluor®647 Goat anti-mouse IgG (1:500; A21235, Invitrogen)
o Manufacturer Validation: Immunofluorescence analysis was performed using HeLa cells stained with alpha Tubulin (236-10501). No nonspecific staining was observed with the secondary antibody alone, or with an isotype control in images captured at 60X magnification. https://www.thermofisher.com/antibody/product/Goat-anti-Mouse-IgG-H-L-Cross-Adsorbed-Secondary-Antibody-Polyclonal/A-21235

Figure 2F
Primary antibodies
o Anti-a-syn (1 ug/ml; Mouse monoclonal, clone SYN 211, Invitrogen, 32-8100)
o In-lab Validation: Antibody tested in rat primary cortical neurons by Western blotting as described in the methods section with non-transduced negative controls.
o Manufacturer Validation: Antibody specificity was demonstrated by CRISPR-Cas9 mediated knockout of target protein. A loss of

signal was observed for target protein in SNCA (KO) cell line compared to control cell line using Anti-SNCA Polyclonal Antibody (Product # 32-8100). https://www.thermofisher.com/antibody/product/alpha-Synuclein-Antibody-clone-Syn-211-Monoclonal/32-8100
o Anti-GFP (1:2000; Rabbit polyclonal, ThermoFisher Scientific, A11122)
o Manufacturer Validation: Antibody specificity was demonstrated by detection of different targets fused to GFP tag in transiently transfected lysates tested. Relative detection of GFP tag was observed across different proteins fused with GFP in H3-GFP and p65-GFP. GFP-variant, YFP is also detected in His-p65-YFP lysate in Western Blot. https://www.thermofisher.com/antibody/product/GFP-Antibody-Polyclonal/A-11122
o Anti β-Actin (1:1000; Mouse monoclonal, clone 8H10D10, Cell Signaling Technologies, 3700)
o Manufacturer Validation: Antibody specificity was demonstrated by western blot analysis of extracts from various cell types as well as western blot analysis of recombinant Actin isoforms using a Pan-Actin Antibody #4968. https://www.cellsignal.com/products/primary-antibodies/b-actin-8h10d10-mouse-mab/3700
Secondary antibodies
o Peroxidase AffiniPure Donkey Anti-Rabbit IgG (1:5000, AB_10015282, 711-035-152, Jackson ImmunoResearch Inc.)
o Manufacturer Validation: The antibody has been tested by ELISA and/or solid-phase adsorbed to ensure minimal cross-reaction with bovine, chicken, goat, guinea pig, syrian hamster, horse, human, mouse, rat and sheep serum proteins, but it may cross-react with immunoglobulins from other species. https://www.jacksonimmuno.com/catalog/products/711-035-152
o Peroxidase AffiniPure Goat Anti-Mouse IgG (1:5000, AB_2338512, 115-035-174, Jackson ImmunoResearch Inc.)
o Manufacturer Validation: The antibody does not react with the heavy chain of mouse IgG. The antibody has been tested by ELISA to ensure minimal cross-reaction with bovine, goat, horse, human, rabbit, rat and sheep immunoglobulins, but it may cross-react with immunoglobulins from other species. https://www.jacksonimmuno.com/catalog/products/115-035-174

Figure 2H
Primary antibodies
o Anti-a-syn (1:500; Mouse monoclonal, clone SYN 211, Invitrogen, 32-8100)
o In-lab Validation: Antibody tested in rat primary cortical neurons and rat brain tissue as described in the methods section.
o Manufacturer Validation: Antibody specificity was demonstrated by CRISPR-Cas9 mediated knockout of target protein. A loss of signal was observed for target protein in SNCA (KO) cell line compared to control cell line using Anti-SNCA Polyclonal Antibody (Product # 32-8100). https://www.thermofisher.com/antibody/product/alpha-Synuclein-Antibody-clone-Syn-211-Monoclonal/32-8100
Secondary antibodies
o AlexaFluor®647 Goat anti-mouse IgG (1:500; A21235, Invitrogen)
o Manufacturer Validation: Immunofluorescence analysis was performed using HeLa cells stained with alpha Tubulin (236-10501). No nonspecific staining was observed with the secondary antibody alone, or with an isotype control in images captured at 60X magnification. https://www.thermofisher.com/antibody/product/Goat-anti-Mouse-IgG-H-L-Cross-Adsorbed-Secondary-Antibody-Polyclonal/A-21235

Figure 2M
Primary antibodies
o Anti-pSer129a-syn (1:1000; Rabbit monoclonal, clone EP1536Y, Abcam, ab51253)
o In-lab validation: Antibody specificity tested on striatum sections from rodent brain injected with synuclein pre-formed fibrils and negative control (secondary only) in rat cortical neurons.
o Manufacturer Validation: This antibody only detects alpha synuclein phosphorylated on Ser129. IHC-P: This antibody showed no staining in human hippocampus normal brain and showed staining in Parkinson's brain as expected. https://www.abcam.com/alpha-synuclein-phospho-s129-antibody-ep1536y-ab51253.html
o Anti-NeuN (1:1000; Rat monoclonal, clone EPR12763, Abcam, ab279297)
o Manufacturer Validation: Tested by Western blotting in human, mouse and rat brain tissue lysate, by immunohistochemistry in human cerebral cortex, by flow cytometry in rat primary neural/glia cells and by immunocytochemistry in SHSY5Y cells. https://www.abcam.com/neun-antibody-epr12763-rat-igg2a-ab279297.html
o Anti-GFP (1:2000; Mouse monoclonal, clone 3E6, Invitrogen, A11120)
o In-lab Validation: Antibody tested in rat primary cortical neurons as described in the methods section.
o Manufacturer Validation: Antibody specificity was demonstrated by detection of different targets fused to GFP tag in transiently transfected lysates tested. Relative detection of GFP tag was observed across different proteins fused with GFP in H3-GFP and p65-GFP in Western Blot. https://www.thermofisher.com/antibody/product/GFP-Antibody-clone-3E6-Monoclonal/A-11120
Secondary antibodies
o AlexaFluor®555 Goat anti-rabbit IgG (1:500; A21429, Invitrogen)
o Manufacturer Validation: Immunofluorescence analysis was performed using HepG2 cells stained with alpha-1 antitrypsin (PA5-16661). No nonspecific staining was observed with the secondary antibody alone, or with an isotype control in images captured at 60X magnification. https://www.thermofisher.com/antibody/product/Goat-anti-Rabbit-IgG-H-L-Highly-Cross-Adsorbed-Secondary-Antibody-Polyclonal/A-21429
o AlexaFluor®488 Goat anti-mouse IgG (1:500, A21131, Invitrogen)
o Manufacturer Validation: Immunofluorescence analysis performed using HeLa cells stained with XRCC1 (MA1-12640). No nonspecific staining observed with the secondary antibody alone or with an isotype control in images captured at 60X magnification. https://www.thermofisher.com/antibody/product/Goat-anti-Mouse-IgG2a-Cross-Adsorbed-Secondary-Antibody-Polyclonal/A-21131
o AlexaFluor®405 Goat anti-rat IgG (1:500, A48261, Invitrogen)
o Manufacturer Validation: This antibody binds to heavy chains on rat IgG and light chains on all rat immunoglobulins. This antibody does not bind non-immunoglobulin rat serum proteins or serum proteins/IgG from mouse, horse, rabbit, bovine, or human. https://www.thermofisher.com/antibody/product/Goat-anti-Rat-IgG-H-L-Highly-Cross-Adsorbed-Secondary-Antibody-Polyclonal/A48261

Figure 3A
Primary antibodies
o Anti-a-syn (1:1000, Mouse monoclonal, clone 42, BD Biosciences, 610787)
o Manufacturer Validation: Specificity routinely tested by western blot analysis. https://www.bdbiosciences.com/en-ca/products/reagents/microscopy-imaging-reagents/immunofluorescence-reagents/purified-mouse-anti-synuclein.610787
o Anti-GFP (1:2000; Rabbit polyclonal, Abcam, ab290)
o In-lab Validation: Antibody tested in non-transduced or non-transfected negative control HEK293 cells.

o Manufacturer Validation: Antibody specificity was demonstrated by detecting the GFP fraction from cell extracts expressing recombinant GFP fusion proteins and has also been shown to be useful on mouse sections fixed with formalin. https://www.abcam.com/gfp-antibody-ab290.html
o Anti-Flag HRP (1:1000; Mouse mAb, Sigma, A8592)
o In-lab Validation: Antibody tested in non-transduced or non-transfected negative control HEK293 cells.
o Manufacturer Validation: Specificity routinely tested by western blot analysis. https://www.sigmaaldrich.com/CA/en/product/sigma/a8592
Secondary antibodies
o Anti-mouse HRP-linked (1:5000; Cell Signaling, 7076)
o Manufacturer Validation: This product is thoroughly validated with CST primary antibodies and will work optimally with the CST western immunoblotting protocol, ensuring accurate and reproducible results. https://www.cellsignal.com/products/secondary-antibodies/anti-mouse-igg-hrp-linked-antibody/7076
o Anti-rabbit HRP-linked (1:5000; Cell Signaling, 7074)
o Manufacturer Validation: This product is thoroughly validated with CST primary antibodies and will work optimally with the CST western immunoblotting protocol, ensuring accurate and reproducible results. https://www.cellsignal.com/products/secondary-antibodies/anti-rabbit-igg-hrp-linked-antibody/7074

Figure 3C
Primary antibodies
o Anti-a-syn (1:500, Mouse monoclonal, BD Biosciences, 610787, clone 42)
o Manufacturer Validation: Specificity routinely tested for immunofluorescence during development. https://www.bdbiosciences.com/en-ca/products/reagents/microscopy-imaging-reagents/immunofluorescence-reagents/purified-mouse-anti-synuclein.610787
o Anti-LAMP1 (1:500; Rabbit polyclonal, Abcam, ab24170)
o Manufacturer Validation: Antibody specificity was demonstrated by Western blotting in Jurkat (Human) whole cell lysate and HEK293 (Human) whole cell lysate, generating a predicted band at 120 kDA. LAMP1 immunohistochemical reactivity is also shown in human cortex sections. Predicted to work with: Mouse, Rat, Chicken, Hamster, Cow, Cat, Dog, Xenopus laevis, Monkey, Zebrafish, African green monkey. https://www.abcam.com/lamp1-antibody-lysosome-marker-ab24170.html
Secondary antibodies
o AlexaFluor®555 Goat anti-rabbit IgG (1:500; A21429, Invitrogen)
o Manufacturer Validation: Immunofluorescence analysis was performed using HepG2 cells stained with alpha-1 antitrypsin (PA5-16661). No nonspecific staining was observed with the secondary antibody alone, or with an isotype control in images captured at 60X magnification. https://www.thermofisher.com/antibody/product/Goat-anti-Rabbit-IgG-H-L-Highly-Cross-Adsorbed-Secondary-Antibody-Polyclonal/A-21429
o AlexaFluor®405 Goat anti-mouse IgG (1:500; 35501BID, Invitrogen)
o Manufacturer Validation: Immunofluorescence analysis of Goat anti-Mouse IgG (H+L) Secondary Antibody, DyLight 405 (Product # 35501BID) was performed using HeLa cells stained with alpha Tubulin (236-10501) Mouse Monoclonal Antibody (Product # A11126). https://www.thermofisher.com/antibody/product/Goat-anti-Mouse-IgG-H-L-Secondary-Antibody-Polyclonal/35501BID

Figure 3D
Primary antibodies
o Anti-LAMP1 (1:500; Rabbit polyclonal, Abcam, ab24170)
o Manufacturer Validation: Antibody specificity was demonstrated by Western blotting in Jurkat (Human) whole cell lysate and HEK293 (Human) whole cell lysate, generating a predicted band at 120 kDA. LAMP1 immunohistochemical reactivity is also shown in human cortex sections. Predicted to work with: Mouse, Rat, Chicken, Hamster, Cow, Cat, Dog, Xenopus laevis, Monkey, Zebrafish, African green monkey. https://www.abcam.com/lamp1-antibody-lysosome-marker-ab24170.html
o Anti-a-syn (1:1000, Mouse monoclonal, clone 42, BD Biosciences, 610787)
o Manufacturer Validation: Specificity routinely tested by western blot analysis. https://www.bdbiosciences.com/en-ca/products/reagents/microscopy-imaging-reagents/immunofluorescence-reagents/purified-mouse-anti-synuclein.610787
o Anti-GFP (1:2000; Rabbit polyclonal, Abcam, ab290)
o In-lab Validation: Antibody tested in non-transduced or non-transfected negative control HEK293 cells.
o Manufacturer Validation: Antibody specificity was demonstrated by detecting the GFP fraction from cell extracts expressing recombinant GFP fusion proteins and has also been shown to be useful on mouse sections fixed with formalin. https://www.abcam.com/gfp-antibody-ab290.html
o Anti β-Actin (1:1000; Rabbit monoclonal clone 13E5, Cell Signaling Technologies, 4970)
o Manufacturer Validation: Antibody specificity was demonstrated by western blot analysis of extracts from various cell types as well as western blot analysis of recombinant Actin isoforms using a Pan-Actin Antibody #4968. https://www.cellsignal.com/products/primary-antibodies/b-actin-13e5-rabbit-mab/4970
Secondary antibodies
o Anti-mouse HRP-linked (1:5000; Cell Signaling, 7076)
o Manufacturer Validation: This product is thoroughly validated with CST primary antibodies and will work optimally with the CST western immunoblotting protocol, ensuring accurate and reproducible results. https://www.cellsignal.com/products/secondary-antibodies/anti-mouse-igg-hrp-linked-antibody/7076
o Anti-rabbit HRP-linked (1:5000; Cell Signaling, 7074)
o Manufacturer Validation: This product is thoroughly validated with CST primary antibodies and will work optimally with the CST western immunoblotting protocol, ensuring accurate and reproducible results. https://www.cellsignal.com/products/secondary-antibodies/anti-rabbit-igg-hrp-linked-antibody/7074

Figure 3E
Primary antibodies
o Anti-CD63 (1:1000; Rabbit monoclonal, clone 2585J, R&D Systems, MAB50482-SP)
o Manufacturer Validation: Antibody specificity was demonstrated by Western blot from lysates of human platelets. A specific band was detected for CD63 at approximately 55 kDa (as indicated). This experiment was conducted under reducing conditions and using Immunoblot Buffer Group 1. https://www.rndsystems.com/products/human-cd63-antibody-2585j_mab50482
o Anti β-Actin (1:1000; Rabbit monoclonal clone 13E5, Cell Signaling Technologies, 4970)
o Manufacturer Validation: Antibody specificity was demonstrated by western blot analysis of extracts from various cell types as

well as western blot analysis of recombinant Actin isoforms using a Pan-Actin Antibody #4968. https://www.cellsignal.com/products/primary-antibodies/b-actin-13e5-rabbit-mab/4970
Secondary antibodies
o Anti-rabbit HRP-linked (1:5000; Cell Signaling, 7074)
o Manufacturer Validation: This product is thoroughly validated with CST primary antibodies and will work optimally with the CST western immunoblotting protocol, ensuring accurate and reproducible results. https://www.cellsignal.com/products/secondary-antibodies/anti-rabbit-igg-hrp-linked-antibody/7074

Figure 3H
Primary antibodies
o Anti-LAMP1 (1:500; Rabbit polyclonal, Abcam, ab24170)
o In-lab Validation: Antibody tested in rat primary cortical neurons and rat brain tissue as described in the methods section.
o Manufacturer Validation: Antibody specificity was demonstrated by Western blotting in Jurkat (Human) whole cell lysate and HEK293 (Human) whole cell lysate, generating a predicted band at 120 kDA. LAMP1 immunohistochemical reactivity is also shown in human cortex sections. Predicted to work with: Mouse, Rat, Chicken, Hamster, Cow, Cat, Dog, Xenopus laevis, Monkey, Zebrafish, African green monkey. https://www.abcam.com/lamp1-antibody-lysosome-marker-ab24170.html
o Anti-a-syn (1:500; Mouse monoclonal, clone SYN 211, Invitrogen, 32-8100)
o In-lab Validation: Antibody tested in rat primary cortical neurons and rat brain tissue as described in the methods section.
o Manufacturer Validation: Antibody specificity was demonstrated by CRISPR-Cas9 mediated knockout of target protein. A loss of signal was observed for target protein in SNCA (KO) cell line compared to control cell line using Anti-SNCA Polyclonal Antibody (Product # 32-8100). https://www.thermofisher.com/antibody/product/alpha-Synuclein-Antibody-clone-Syn-211-Monoclonal/32-8100
Secondary antibodies
o AlexaFluor®488 Goat anti-rabbit IgG (1:500; A32731, Invitrogen)
o Manufacturer Validation: Anti-rabbit IgG whole antibodies are pre cross-adsorbed against bovine IgG, goat IgG, mouse IgG, rat IgG, and human IgG to increase specificity of the antibody resulting in less background staining and cross-reactivity. https://www.thermofisher.com/antibody/product/Goat-anti-Rabbit-IgG-H-L-Highly-Cross-Adsorbed-Secondary-Antibody-Polyclonal/A32731
o AlexaFluor®647 Goat anti-mouse IgG (1:500; A21235, Invitrogen)
o Manufacturer Validation: Immunofluorescence analysis was performed using HeLa cells stained with alpha Tubulin (236-10501). No nonspecific staining was observed with the secondary antibody alone, or with an isotype control in images captured at 60X magnification. https://www.thermofisher.com/antibody/product/Goat-anti-Mouse-IgG-H-L-Cross-Adsorbed-Secondary-Antibody-Polyclonal/A-21235

Figure 4C
Primary antibodies
o Anti-TH (1:500; Rabbit polyclonal, Invitrogen, P21962)
o In-lab Validation: Antibody tested in iPSC-derived dopaminergic neurons and rat brain tissue as described in the methods section.
o Manufacturer Validation: Immunofluorescence analysis of DA in H9 ESCs differentiated with PSC Dopaminergic neuron differentiation kit. H9 ESCs were specified to become midbrain floor plate (FP) progenitors which were further expanded and cryopreserved. Recovered FP progenitors were then matured for additional 14 days. https://www.thermofisher.com/antibody/product/Tyrosine-Hydroxylase-Antibody-Polyclonal/P21962
Secondary antibodies
o AlexaFluor®647 Goat anti-rabbit IgG (1:500; A21245, Invitrogen)
o Manufacturer Validation: Immunofluorescence analysis was performed using HeLa cells stained with alpha Tubulin (PA5-16891). No nonspecific staining was observed with the secondary antibody alone, or with an isotype control in images captured at 60X magnification. https://www.thermofisher.com/antibody/product/Goat-anti-Rabbit-IgG-H-L-Highly-Cross-Adsorbed-Secondary-Antibody-Polyclonal/A-21245

Figure 5B/5F
Primary antibodies
o Anti-TH (1:500; Rabbit polyclonal, Invitrogen, P21962)
o In-lab Validation: Antibody tested in iPSC-derived dopaminergic neurons and rat brain tissue as described in the methods section.
o Manufacturer Validation: Immunofluorescence analysis of DA in H9 ESCs differentiated with PSC Dopaminergic neuron differentiation kit. H9 ESCs were specified to become midbrain floor plate (FP) progenitors which were further expanded and cryopreserved. Recovered FP progenitors were then matured for additional 14 days. https://www.thermofisher.com/antibody/product/Tyrosine-Hydroxylase-Antibody-Polyclonal/P21962
o Anti-a-syn (1:500; Mouse monoclonal, clone SYN 211, Invitrogen, 32-8100)
o In-lab Validation: Antibody tested in rat primary cortical neurons and rat brain tissue as described in the methods section.
o Manufacturer Validation: Antibody specificity was demonstrated by CRISPR-Cas9 mediated knockout of target protein. A loss of signal was observed for target protein in SNCA (KO) cell line compared to control cell line using Anti-SNCA Polyclonal Antibody (Product # 32-8100). https://www.thermofisher.com/antibody/product/alpha-Synuclein-Antibody-clone-Syn-211-Monoclonal/32-8100
Secondary antibodies
o AlexaFluor®555 Goat anti-rabbit IgG (1:500; Invitrogen, A21429)
o Manufacturer Validation: Immunofluorescence analysis was performed using HepG2 cells stained with alpha-1 antitrypsin (PA5-16661). No nonspecific staining was observed with the secondary antibody alone, or with an isotype control in images captured at 60X magnification. https://www.thermofisher.com/antibody/product/Goat-anti-Rabbit-IgG-H-L-Highly-Cross-Adsorbed-Secondary-Antibody-Polyclonal/A-21429
o AlexaFluor®647 Goat anti-mouse IgG (1:500; Invitrogen, A21235)
o Manufacturer Validation: Immunofluorescence analysis was performed using HeLa cells stained with alpha Tubulin (236-10501). No nonspecific staining was observed with the secondary antibody alone, or with an isotype control in images captured at 60X magnification. https://www.thermofisher.com/antibody/product/Goat-anti-Mouse-IgG-H-L-Cross-Adsorbed-Secondary-Antibody-Polyclonal/A-21235

Figure 6A
Primary antibodies

o Anti-a-syn (1:500; Mouse monoclonal, clone SYN 211, Invitrogen, 32-8100)
o In-lab Validation: Antibody tested in rat primary cortical neurons and rat brain tissue as described in the methods section.
o Manufacturer Validation: Antibody specificity was demonstrated by CRISPR-Cas9 mediated knockout of target protein. A loss of signal was observed for target protein in SNCA (KO) cell line compared to control cell line using Anti-SNCA Polyclonal Antibody (Product # 32-8100). https://www.thermofisher.com/antibody/product/alpha-Synuclein-Antibody-clone-Syn-211-Monoclonal/32-8100
Secondary antibodies
o AlexaFluor®647 Goat anti-mouse IgG (1:500; Invitrogen, A21235)
o Manufacturer Validation: Immunofluorescence analysis was performed using HeLa cells stained with alpha Tubulin (236-10501). No nonspecific staining was observed with the secondary antibody alone, or with an isotype control in images captured at 60X magnification. https://www.thermofisher.com/antibody/product/Goat-anti-Mouse-IgG-H-L-Cross-Adsorbed-Secondary-Antibody-Polyclonal/A-21235

Figure 6D
Primary antibodies
o Anti-TH (1:500, Sheep monoclonal, clone LNC1, Chemicon, MAB318)
o In-lab Validation: Antibody tested in iPSC-derived dopaminergic neurons and rat brain tissue as described in the methods section.
o Manufacturer Validation: Recognizes an epitope on the outside of the regulatory N-terminus. Recognizes a protein of approximately 59-61 kDa by Western blot. Does not react with the following on Western Blots: dopamine-beta-hydroxylase, phenylalanine hydroxylase, trytophan hydroxylase, dehydropteridine reductase, sepiapterin reductase or phenethanolamine-N-methyl transferase (PNMT). https://www.emdmillipore.com/CA/en/product/Anti-Tyrosine-Hydroxylase-Antibody-clone-LNC1,MM_NF-MAB318
o Anti-LAMP1 (1:500; Rabbit polyclonal, Abcam, ab24170)
o In-lab Validation: Antibody tested in rat primary cortical neurons and rat brain tissue as described in the methods section.
o Manufacturer Validation: Antibody specificity was demonstrated by Western blotting in Jurkat (Human) whole cell lysate and HEK293 (Human) whole cell lysate, generating a predicted band at 120 kDA. LAMP1 immunohistochemical reactivity is also shown in human cortex sections. Predicted to work with: Mouse, Rat, Chicken, Hamster, Cow, Cat, Dog, Xenopus laevis, Monkey, Zebrafish, African green monkey. https://www.abcam.com/lamp1-antibody-lysosome-marker-ab24170.html
o Anti-a-syn (1:500; Mouse monoclonal, clone SYN 211, Invitrogen, 32-8100)
o In-lab Validation: Antibody tested in rat primary cortical neurons and rat brain tissue as described in the methods section.
o Manufacturer Validation: Antibody specificity was demonstrated by CRISPR-Cas9 mediated knockout of target protein. A loss of signal was observed for target protein in SNCA (KO) cell line compared to control cell line using Anti-SNCA Polyclonal Antibody (Product # 32-8100). https://www.thermofisher.com/antibody/product/alpha-Synuclein-Antibody-clone-Syn-211-Monoclonal/32-8100
Secondary antibodies
o AlexaFluor®405 Goat anti-rabbit IgG (1:500; Invitrogen, A48254)
o Manufacturer Validation: This antibody binds to heavy chains on rabbit IgG and light chains on all rabbit immunoglobulins. This antibody does not bind to human, mouse or rat serum proteins/IgG or rabbit non-immunoglobulin serum proteins. https://www.thermofisher.com/antibody/product/Goat-anti-Rabbit-IgG-H-L-Highly-Cross-Adsorbed-Secondary-Antibody-Polyclonal/A48254
o AlexaFluor®488 Donkey anti-sheep IgG (1:500; Invitrogen, A11015)
o Manufacturer Validation: To minimize cross-reactivity, these donkey anti-sheep IgG (H+L) whole secondary antibodies have been affinity purified and cross-adsorbed against mouse, rabbit, bovine, and human sera, and human IgG. https://www.thermofisher.com/antibody/product/Donkey-anti-Sheep-IgG-H-L-Cross-Adsorbed-Secondary-Antibody-Polyclonal/A-11015
o AlexaFluor®647 Goat anti-mouse IgG (1:500; Invitrogen, A21235)
o Manufacturer Validation: Immunofluorescence analysis was performed using HeLa cells stained with alpha Tubulin (236-10501). No nonspecific staining was observed with the secondary antibody alone, or with an isotype control in images captured at 60X magnification. https://www.thermofisher.com/antibody/product/Goat-anti-Mouse-IgG-H-L-Cross-Adsorbed-Secondary-Antibody-Polyclonal/A-21235

Figure 6G
Primary antibodies
o Anti-oligomeric synuclein (1:5000; Mouse monoclonal, clone SYN-O2, Courtesy of El-Agnaf lab)
o In-lab Validation: Validated in rat substantia nigra tissue injected with AAV1/2 mutant A53T synuclein. Specifically recognizes soluble oligomers and late aggregates “amyloid fibrils” of α-syn and has a high binding affinity for o-α-syn. It exclusively bound to α-syn aggregates (amyloid fibrils and soluble oligomers) without cross-reactivity with monomers or fibrils generated from other amyloid proteins (https://www.ncbi.nlm.nih.gov/pmc/articles/PMC4717559/)
o Anti-TH (1:500; Chicken polyclonal, Abcam, ab76442)
o In-lab Validation: Antibody tested in iPSC-derived dopaminergic neurons and rat brain tissue as described in the methods section.
o Manufacturer Validation: Immunohistochemistry analysis of hippocampus and rostral ventral lateral medulla tissue in adult mouse brain labelling tyrosine hydroxylase. https://www.abcam.com/tyrosine-hydroxylase-antibody-ab76442.html?productWallTab=ShowAll
o Anti-RFP (1:1000; Rabbit polyclonal, Rockland, 600-401-379)
o In-lab Validation: Antibody tested in iPSC-derived dopaminergic neurons as described in the methods section with no primary (secondary only) negative controls.
o Manufacturer Validation: Antibody specificity to RFP and its variants tested by ELISA, western blot, IF, and IHC. Assay by immunoelectrophoresis resulted in a single precipitin arc against anti-Rabbit Serum and purified and partially purified Red Fluorescent Protein (Discosoma). No reaction was observed against Human, Mouse or Rat serum proteins. https://www.rockland.com/categories/primary-antibodies/rfp-antibody-pre-adsorbed-600-401-379/
Secondary antibodies
o Biotinylated Goat anti-mouse IgG (1:500; Jackson ImmunoResearch Inc, AB_2338567, 115-065-146)
o Manufacturer Validation: Based on immunoelectrophoresis and/or ELISA, the antibody reacts with whole molecule mouse IgG. It also reacts with the light chains of other mouse immunoglobulins. No antibody was detected against non-immunoglobulin serum proteins. The antibody has been tested by ELISA and/or solid-phase adsorbed to ensure minimal cross-reaction with human, bovine, horse, rabbit and swine serum proteins, but it may cross-react with immunoglobulins from other species. https://www.jacksonimmuno.com/catalog/products/115-065-146. Used in combination with AlexaFluor®488 Streptavidin.
o AlexaFluor®555 Goat anti-rabbit IgG (1:500; Invitrogen, A21429)

o Manufacturer Validation: Immunofluorescence analysis was performed using HepG2 cells stained with alpha-1 antitrypsin (PA5-16661). No nonspecific staining was observed with the secondary antibody alone, or with an isotype control in images captured at 60X magnification. https://www.thermofisher.com/antibody/product/Goat-anti-Rabbit-IgG-H-L-Highly-Cross-Adsorbed-Secondary-Antibody-Polyclonal/A-21429
o AlexaFluor®647 Goat anti-chicken IgG (1:500; Invitrogen, A32933)
o Manufacturer Validation: Specificity of secondary antibody was demonstrated by specific detection of the target immunoglobulin in Western Blot. Antibody specificity was demonstrated by specific detection of Chicken IgY but not in other species. https://www.thermofisher.com/antibody/product/Goat-anti-Chicken-IgY-H-L-Cross-Adsorbed-Secondary-Antibody-Polyclonal/A32933

Figure S1D
Primary antibodies
o Anti-HA (1:2000, Mouse monoclonal, clone F-7, Santa Cruz, sc-7392)
o In-lab Validation: Antibody tested in non-transfected negative control HEK293 cells.
o Manufacturer Validation: Specificity tested by Western blot analysis of HA-tagged fusion proteins showing N-terminal HA-tagged JNK2 and JNK1 and C-terminal HA-tagged Daxx. https://www.scbt.com/p/ha-probe-antibody-f-7
o Anti-GFP (1:2000; Rabbit polyclonal, Abcam, ab290)
o In-lab Validation: Antibody tested in non-transduced or non-transfected negative control HEK293 cells.
o Manufacturer Validation: Antibody specificity was demonstrated by detecting the GFP fraction from cell extracts expressing recombinant GFP fusion proteins and has also been shown to be useful on mouse sections fixed with formalin. https://www.abcam.com/gfp-antibody-ab290.html
o Anti-Flag HRP (1:1000; Mouse monoclonal, Sigma, A8592)
o In-lab Validation: Antibody tested in non-transduced or non-transfected negative control HEK293 cells.
o Manufacturer Validation: Specificity routinely tested by western blot analysis. https://www.sigmaaldrich.com/CA/en/product/sigma/a8592
Secondary antibodies
o Anti-mouse HRP-linked (1:5000; Cell Signaling, 7076)
o Manufacturer Validation: This product is thoroughly validated with CST primary antibodies and will work optimally with the CST western immunoblotting protocol, ensuring accurate and reproducible results. https://www.cellsignal.com/products/secondary-antibodies/anti-mouse-igg-hrp-linked-antibody/7076
o Anti-rabbit HRP-linked (1:5000; Cell Signaling, 7074)
o Manufacturer Validation: This product is thoroughly validated with CST primary antibodies and will work optimally with the CST western immunoblotting protocol, ensuring accurate and reproducible results. https://www.cellsignal.com/products/secondary-antibodies/anti-rabbit-igg-hrp-linked-antibody/7074

Figure S2A
Primary antibodies
o Anti-a-syn (1:1000, Mouse monoclonal, clone 42, BD Biosciences, 610787)
o Manufacturer Validation: Specificity routinely tested by western blot analysis. https://www.bdbiosciences.com/en-ca/products/reagents/microscopy-imaging-reagents/immunofluorescence-reagents/purified-mouse-anti-synuclein.610787
o Anti-GFP (1:2000; Rabbit polyclonal, Abcam, ab290)
o In-lab Validation: Antibody tested in non-transduced or non-transfected negative control HEK293 cells.
o Manufacturer Validation: Antibody specificity was demonstrated by detecting the GFP fraction from cell extracts expressing recombinant GFP fusion proteins and has also been shown to be useful on mouse sections fixed with formalin. https://www.abcam.com/gfp-antibody-ab290.html
o Anti-Flag HRP (1:1000; Mouse mAb, Sigma, A8592)
o In-lab Validation: Antibody tested in non-transduced or non-transfected negative control HEK293 cells.
o Manufacturer Validation: Specificity routinely tested by western blot analysis. https://www.sigmaaldrich.com/CA/en/product/sigma/a8592
Secondary antibodies
o Anti-mouse HRP-linked (1:5000; Cell Signaling, 7076)
o Manufacturer Validation: This product is thoroughly validated with CST primary antibodies and will work optimally with the CST western immunoblotting protocol, ensuring accurate and reproducible results. https://www.cellsignal.com/products/secondary-antibodies/anti-mouse-igg-hrp-linked-antibody/7076
o Anti-rabbit HRP-linked (1:5000; Cell Signaling, 7074)
o Manufacturer Validation: This product is thoroughly validated with CST primary antibodies and will work optimally with the CST western immunoblotting protocol, ensuring accurate and reproducible results. https://www.cellsignal.com/products/secondary-antibodies/anti-rabbit-igg-hrp-linked-antibody/7074

Figure S2C
Primary antibodies
o Anti-a-syn (1:1000, Mouse monoclonal, clone 42, BD Biosciences, 610787)
o Manufacturer Validation: Specificity routinely tested by western blot analysis. https://www.bdbiosciences.com/en-ca/products/reagents/microscopy-imaging-reagents/immunofluorescence-reagents/purified-mouse-anti-synuclein.610787
o Anti-Flag HRP (1:1000; Mouse monoclonal, Sigma, A8592)
o In-lab Validation: Antibody tested in non-transduced or non-transfected negative control HEK293 cells.
o Manufacturer Validation: Specificity routinely tested by western blot analysis. https://www.sigmaaldrich.com/CA/en/product/sigma/a8592
Secondary antibodies
o Anti-mouse HRP-linked (1:5000; Cell Signaling, 7076)
o Manufacturer Validation: This product is thoroughly validated with CST primary antibodies and will work optimally with the CST western immunoblotting protocol, ensuring accurate and reproducible results. https://www.cellsignal.com/products/secondary-antibodies/anti-mouse-igg-hrp-linked-antibody/7076

Figure S2D/S2E
Primary antibodies
o Anti-a-syn (1:500, Mouse monoclonal, clone 42, BD Biosciences, 610787)

o In-lab Validation: Specificity tested in rat primary cortical neurons by Western blotting and immunocytochemistry.
o Manufacturer Validation: Specificity routinely tested by western blot analysis. https://www.bdbiosciences.com/en-ca/products/reagents/microscopy-imaging-reagents/immunofluorescence-reagents/purified-mouse-anti-synuclein.610787
o Anti-CHMP2B (1:50; Rabbit polyclonal, Abcam, ab33174)
o In-lab Validation: Antibody tested in rat primary cortical neurons as described in the methods section.
o Manufacturer Validation: Antibody specificity tested in wild-type and CHMP2B knockout samples subjected to SDS-PAGE. Antibody shown to recognize CHMP2B in wild-type A549 cells, but not at expected MW in CHMP2B knockout cells. Additional cross-reactive bands were observed in the wild-type and knockout cells. https://www.abcam.com/chmp2b-antibody-ab33174.html

Figure S3D
Primary antibodies
o Anti-a-syn (1:1000, Mouse monoclonal, clone 42, BD Biosciences, 610787)
o Manufacturer Validation: Specificity routinely tested by western blot analysis. https://www.bdbiosciences.com/en-ca/products/reagents/microscopy-imaging-reagents/immunofluorescence-reagents/purified-mouse-anti-synuclein.610787
o Anti β-Actin (1:1000; Rabbit monoclonal, clone 13E5, Cell Signaling Technologies, 4970)
o Manufacturer Validation: Antibody specificity was demonstrated by western blot analysis of extracts from various cell types as well as western blot analysis of recombinant Actin isoforms using a Pan-Actin Antibody #4968. https://www.cellsignal.com/products/primary-antibodies/b-actin-13e5-rabbit-mab/4970
Secondary antibodies
o Anti-mouse HRP-linked (1:5000; Cell Signaling, 7076)
o Manufacturer Validation: This product is thoroughly validated with CST primary antibodies and will work optimally with the CST western immunoblotting protocol, ensuring accurate and reproducible results. https://www.cellsignal.com/products/secondary-antibodies/anti-mouse-igg-hrp-linked-antibody/7076
o Anti-rabbit HRP-linked (1:5000; Cell Signaling, 7074)
o Manufacturer Validation: This product is thoroughly validated with CST primary antibodies and will work optimally with the CST western immunoblotting protocol, ensuring accurate and reproducible results. https://www.cellsignal.com/products/secondary-antibodies/anti-rabbit-igg-hrp-linked-antibody/7074

Figure S3F
Primary antibodies
o Anti-HA (1:1000, Rat monoclonal, clone 3F10, Roche, 11867423001)
o In-lab Validation: Antibody tested in rat primary cortical neurons as described in the methods section.
o Manufacturer Validation: Detection of native influenza hemagglutinin protein and recombinant proteins that contain the HA epitope tested using Western blot. https://www.sigmaaldrich.com/CA/en/product/roche/roahaha
Secondary antibodies
o AlexaFluor®555 Goat anti-rat IgG (1:500, Invitrogen, A48263)
o Manufacturer Validation: This antibody binds to heavy chains on rat IgG and light chains on all rat immunoglobulins. This antibody does not bind non-immunoglobulin rat serum proteins or serum proteins/IgG from mouse, horse, rabbit, bovine, or human. https://www.thermofisher.com/antibody/product/Goat-anti-Rat-IgG-H-L-Highly-Cross-Adsorbed-Secondary-Antibody-Polyclonal/A48263

Figure S4A
Primary antibodies
o Anti-LAMP1 (1:500; Rabbit polyclonal, Abcam, ab24170)
o Manufacturer Validation: Antibody specificity was demonstrated by Western blotting in Jurkat (Human) whole cell lysate and HEK293 (Human) whole cell lysate, generating a predicted band at 120 kDA. LAMP1 immunohistochemical reactivity is also shown in human cortex sections. Predicted to work with: Mouse, Rat, Chicken, Hamster, Cow, Cat, Dog, Xenopus laevis, Monkey, Zebrafish, African green monkey. https://www.abcam.com/lamp1-antibody-lysosome-marker-ab24170.html
o Anti-a-syn (1:1000, Mouse monoclonal, clone 42, BD Biosciences, 610787)
o Manufacturer Validation: Specificity routinely tested by western blot analysis. https://www.bdbiosciences.com/en-ca/products/reagents/microscopy-imaging-reagents/immunofluorescence-reagents/purified-mouse-anti-synuclein.610787
Secondary antibodies
o Anti-mouse HRP-linked (1:5000; Cell Signaling, 7076)
o Manufacturer Validation: This product is thoroughly validated with CST primary antibodies and will work optimally with the CST western immunoblotting protocol, ensuring accurate and reproducible results. https://www.cellsignal.com/products/secondary-antibodies/anti-mouse-igg-hrp-linked-antibody/7076
o Anti-rabbit HRP-linked (1:5000; Cell Signaling, 7074)
o Manufacturer Validation: This product is thoroughly validated with CST primary antibodies and will work optimally with the CST western immunoblotting protocol, ensuring accurate and reproducible results. https://www.cellsignal.com/products/secondary-antibodies/anti-rabbit-igg-hrp-linked-antibody/7074

Figure S4B
Primary antibodies
o Anti-Rab7 (1:500; Rabbit monoclonal, clone D95F2, Cell Signaling Technology, 9367)
o In-lab Validation: Antibody tested in rat primary cortical neurons as described in the methods section.
o Manufacturer Validation: Antibody specificity was demonstrated by Western blot analysis of extracts from HeLa, C2C12, C6 and COS cells and immunofluorescent analysis of SK-MEL-28 cells. https://www.cellsignal.com/products/primary-antibodies/rab7-d95f2-xp-rabbit-mab/9367?N=3639413825+4294956287&Nrpp=30&No=120&fromPage=plp
o Anti-RFP (1:500; Goat polyclonal, Rockland, 200-101-379)
o In-lab Validation: Antibody tested in rat primary cortical neurons as described in the methods section.
o Manufacturer Validation: Antibody specificity to RFP and its variants tested by SDS-Page and Western blot analysis. Assay by immunoelectrophoresis resulted in a single precipitin arc against anti-Goat Serum and purified and partially purified Red Fluorescent Protein (Discosoma). No reaction was observed against Human, Mouse or Rat serum proteins. https://www.rockland.com/categories/primary-antibodies/rfp-antibody-200-101-379/
o Anti-HA (1:1000, Rat monoclonal, clone 3F10, Roche, 11867423001)
o In-lab Validation: Antibody tested in rat primary cortical neurons as described in the methods section.
o Manufacturer Validation: Detection of native influenza hemagglutinin protein and recombinant proteins that contain the HA

epitope tested using Western blot. https://www.sigmaaldrich.com/CA/en/product/roche/roahaha
Secondary antibodies
o AlexaFluor®488 Goat anti-rabbit IgG (1:500; Invitrogen, A32731)
o Manufacturer Validation: Anti-rabbit IgG whole antibodies are pre cross-adsorbed against bovine IgG, goat IgG, mouse IgG, rat IgG, and human IgG to increase specificity of the antibody resulting in less background staining and cross-reactivity. https://www.thermofisher.com/antibody/product/Goat-anti-Rabbit-IgG-H-L-Highly-Cross-Adsorbed-Secondary-Antibody-Polyclonal/A32731
o AlexaFluor®555 Donkey anti-goat IgG (1:500; Invitrogen, A21432)
o Manufacturer Validation: Donkey anti-goat IgG (H+L) whole secondary antibodies have been affinity purified and cross-adsorbed against rabbit, rat, mouse, and human IgG. https://www.thermofisher.com/antibody/product/Donkey-anti-Goat-IgG-H-L-Cross-Adsorbed-Secondary-Antibody-Polyclonal/A-21432
o AlexaFluor®647 Goat anti-rat IgG (1:500; Invitrogen, A21247)
o Manufacturer Validation: Immunofluorescence analysis was performed using A549 cells stained with alpha Tubulin (YL1/2). No nonspecific staining was observed with the secondary antibody alone, or with an isotype control in images captured at 60X magnification. https://www.thermofisher.com/antibody/product/Goat-anti-Rat-IgG-H-L-Cross-Adsorbed-Secondary-Antibody-Polyclonal/A-21247

Figure S4D
Primary antibodies
o Anti-Cathepsin B (1:100; Goat polyclonal, Novus Biologicals, AF965)
o In-lab Validation: Specificity tested in cortical neurons from rat (92.3% homology with mouse) to optimize antibody dilutions for immunocytochemistry with negative controls (secondary only).
o Manufacturer Validation: Detects mouse Cathepsin B in direct ELISAs and Western blots. In direct ELISAs, less than 5% cross-reactivity with recombinant mouse Cathepsin H is observed. https://www.novusbio.com/products/cathepsin-b-antibody_af965
o Anti-a-syn (1:500; Mouse monoclonal, clone SYN 211, Invitrogen, 32-8100)
o In-lab Validation: Antibody tested in rat primary cortical neurons and rat brain tissue as described in the methods section.
o Manufacturer Validation: Antibody specificity was demonstrated by CRISPR-Cas9 mediated knockout of target protein. A loss of signal was observed for target protein in SNCA (KO) cell line compared to control cell line using Anti-SNCA Polyclonal Antibody (Product # 32-8100). https://www.thermofisher.com/antibody/product/alpha-Synuclein-Antibody-clone-Syn-211-Monoclonal/32-8100
Secondary antibodies
o AlexaFluor®488 Donkey anti-goat IgG (1:4000; Invitrogen, A32814)
o Manufacturer Validation: This antibody binds to heavy chains on goat IgG and light chains on all goat immunoglobulins. This antibody does not bind non-immunoglobulin goat serum proteins or IgG from human, mouse, rabbit or rat. https://www.thermofisher.com/antibody/product/Donkey-anti-Goat-IgG-H-L-Highly-Cross-Adsorbed-Secondary-Antibody-Polyclonal/A32814
o AlexaFluor®647 Donkey anti-mouse IgG (1:500; Invitrogen, A31571)
o Manufacturer Validation: Immunofluorescence analysis was performed using HeLa cells stained with alpha Tubulin (A11126). No nonspecific staining was observed with the secondary antibody alone or with an isotype control in images captured at 60X magnification. https://www.thermofisher.com/antibody/product/Donkey-anti-Mouse-IgG-H-L-Highly-Cross-Adsorbed-Secondary-Antibody-Polyclonal/A-31571

Figure S4F
Primary antibodies
o Anti-Cathepsin D (1:250; Rat monoclonal, clone 204712, Novus Biologicals, MAB1029)
o In-lab Validation: Specificity tested in cortical neurons from rat (90.9% homology with mouse) to optimize antibody dilutions for immunocytochemistry with negative controls (secondary only).
o Manufacturer Validation: Detects both the pro and active forms of mouse Cathepsin D in direct ELISAs and Western blots. In Western blots, no cross-reactivity with recombinant human Cathepsin D or recombinant mouse Cathepsin E is observed. https://www.novusbio.com/products/cathepsin-d-antibody-204712_mab1029
o Anti-a-syn (1:500; Mouse monoclonal, clone SYN 211, Invitrogen, 32-8100)
o In-lab Validation: Antibody tested in rat primary cortical neurons and rat brain tissue as described in the methods section.
o Manufacturer Validation: Antibody specificity was demonstrated by CRISPR-Cas9 mediated knockout of target protein. A loss of signal was observed for target protein in SNCA (KO) cell line compared to control cell line using Anti-SNCA Polyclonal Antibody (Product # 32-8100). https://www.thermofisher.com/antibody/product/alpha-Synuclein-Antibody-clone-Syn-211-Monoclonal/32-8100
Secondary antibodies
o AlexaFluor®488 Goat anti-rat IgG (1:1000; Invitrogen, A11006)
o Manufacturer Validation: Immunofluorescence analysis was performed using A549 cells stained with alpha Tubulin (MA1-80017). No nonspecific staining was observed with the secondary antibody alone, or with an isotype control in images captured at 60X magnification. https://www.thermofisher.com/antibody/product/Goat-anti-Rat-IgG-H-L-Cross-Adsorbed-Secondary-Antibody-Polyclonal/A-11006
o AlexaFluor®647 Goat anti-mouse IgG (1:500; Invitrogen, A21235)
o Manufacturer Validation: Immunofluorescence analysis was performed using HeLa cells stained with alpha Tubulin (236-10501). No nonspecific staining was observed with the secondary antibody alone, or with an isotype control in images captured at 60X magnification. https://www.thermofisher.com/antibody/product/Goat-anti-Mouse-IgG-H-L-Cross-Adsorbed-Secondary-Antibody-Polyclonal/A-21235

Figure S5A
Primary antibodies
o Anti-TH (1:500; Rabbit polyclonal, Invitrogen, P21962)
o In-lab Validation: Antibody tested in iPSC-derived dopaminergic neurons and rat brain tissue as described in the methods section.
o Manufacturer Validation: Immunofluorescence analysis of DA in H9 ESCs differentiated with PSC Dopaminergic neuron differentiation kit. H9 ESCs were specified to become midbrain floor plate (FP) progenitors which were further expanded and cryopreserved. Recovered FP progenitors were then matured for additional 14 days. https://www.thermofisher.com/antibody/product/Tyrosine-Hydroxylase-Antibody-Polyclonal/P21962
Secondary antibodies
o AlexaFluor®647 Goat anti-rabbit IgG (1:500; Invitrogen, A21245)

o Manufacturer Validation: Immunofluorescence analysis was performed using HeLa cells stained with alpha Tubulin (PA5-16891). No nonspecific staining was observed with the secondary antibody alone, or with an isotype control in images captured at 60X magnification. https://www.thermofisher.com/antibody/product/Goat-anti-Rabbit-IgG-H-L-Highly-Cross-Adsorbed-Secondary-Antibody-Polyclonal/A-21245

Figure S5B
Primary antibodies
o Anti-pSer129a-syn (1:1000; Rabbit monoclonal, clone EP1536Y, Abcam, ab51253)
o In-lab validation: Antibody specificity tested on striatum sections from rodent brain injected with synuclein pre-formed fibrils and negative control (secondary only) in rat cortical neurons.
o Manufacturer Validation: This antibody only detects alpha synuclein phosphorylated on Ser129. IHC-P: This antibody showed no staining in human hippocampus normal brain and showed staining in Parkinson's brain as expected. https://www.abcam.com/alpha-synuclein-phospho-s129-antibody-ep1536y-ab51253.html
Secondary antibodies
o AlexaFluor®405 Goat anti-rabbit IgG (1:500; Invitrogen, A48254)
o Manufacturer Validation: This antibody binds to heavy chains on rabbit IgG and light chains on all rabbit immunoglobulins. This antibody does not bind to human, mouse or rat serum proteins/IgG or rabbit non-immunoglobulin serum proteins. https://www.thermofisher.com/antibody/product/Goat-anti-Rabbit-IgG-H-L-Highly-Cross-Adsorbed-Secondary-Antibody-Polyclonal/A48254

Figure S5E
Primary antibodies
o Anti-LAMP1 (1:500; Rabbit polyclonal, Abcam, ab24170)
o In-lab Validation: Antibody tested in rat primary cortical neurons and rat brain tissue as described in the methods section.
o Manufacturer Validation: Antibody specificity was demonstrated by Western blotting in Jurkat (Human) whole cell lysate and HEK293 (Human) whole cell lysate, generating a predicted band at 120 kDA. LAMP1 immunohistochemical reactivity is also shown in human cortex sections. Predicted to work with: Mouse, Rat, Chicken, Hamster, Cow, Cat, Dog, Xenopus laevis, Monkey, Zebrafish, African green monkey. https://www.abcam.com/lamp1-antibody-lysosome-marker-ab24170.html
o Anti-a-syn (1:500; Mouse monoclonal, clone SYN 211, Invitrogen, 32-8100)
o In-lab Validation: Antibody tested in rat primary cortical neurons and rat brain tissue as described in the methods section.
o Manufacturer Validation: Antibody specificity was demonstrated by CRISPR-Cas9 mediated knockout of target protein. A loss of signal was observed for target protein in SNCA (KO) cell line compared to control cell line using Anti-SNCA Polyclonal Antibody (Product # 32-8100). https://www.thermofisher.com/antibody/product/alpha-Synuclein-Antibody-clone-Syn-211-Monoclonal/32-8100
Secondary antibodies
o AlexaFluor®647 Goat anti-mouse IgG (1:500; Invitrogen, A21235)
o Manufacturer Validation: Immunofluorescence analysis was performed using HeLa cells stained with alpha Tubulin (236-10501). No nonspecific staining was observed with the secondary antibody alone, or with an isotype control in images captured at 60X magnification. https://www.thermofisher.com/antibody/product/Goat-anti-Mouse-IgG-H-L-Cross-Adsorbed-Secondary-Antibody-Polyclonal/A-21235
o AlexaFluor®555 Goat anti-rabbit IgG (1:500; Invitrogen, A21429)
o Manufacturer Validation: Immunofluorescence analysis was performed using HepG2 cells stained with alpha-1 antitrypsin (PA5-16661). No nonspecific staining was observed with the secondary antibody alone, or with an isotype control in images captured at 60X magnification. https://www.thermofisher.com/antibody/product/Goat-anti-Rabbit-IgG-H-L-Highly-Cross-Adsorbed-Secondary-Antibody-Polyclonal/A-21429

Validation

Antibodies were chosen based on citations on the manufacturers website and, if necessary, further validated in our labs by including a primary only or secondary only condition or by overexpression for tags or by knocking down of expression of the target protein. Further information is provided in the section "Antibody used" in the Reporting Summary.

Eukaryotic cell lines

Policy information about cell lines and Sex and Gender in Research

Cell line source(s)

HEK293, HEK293T and SHSY5Y cells were from ATCC. SNCA triplication fibroblasts were obtained from Coriell Institute for Medical Research/National Institute of Neurological Disorders and Stroke (NINDS). iPSC-derived dopaminergic neurons were purchased from Fujifilm Cellular Dynamics (#R1109 and #R1088)

Authentication

Cells were not further authenticated outside source.

Mycoplasma contamination

All cell lines were tested negative for mycoplasma contamination.

Commonly misidentified lines
(See ICLAC register)

No commonly misidentified cell lines were used in the study.

Animals and other research organisms

Policy information about studies involving animals; ARRIVE guidelines recommended for reporting animal research, and Sex and Gender in Research

Laboratory animals

Female Sprague Dawley rats aged 6-12 months (weighing approximately 275 g) were used for all rodent experiments. BZ555 [dat-1p::gfp] was obtained from the C. elegans Genetics Center (CGC; University of Minnesota, St Paul, MN, USA), TWH1 ([dat-1p::a-syn(A30P), ges-1p::DsRed]; [dat-1p::gfp]) was obtained by crossing BZ555 with A30P a-syn transgenic animals (kindly provided by Dr.

	Takeshi Iwatsubo, University of Tokyo). Adult hermaphrodites were used for all C. elegans experiments.
Wild animals	This study did not involve wild animals.
Reporting on sex	This study used preclinical models of more than one sex: 1) female and male human cells harboring disease-causing mutations, 2) hermaphroditic C. elegans, 3) female Sprague Dawley rats.
Field-collected samples	This study did not involve field-collected samples.
Ethics oversight	All procedures were approved by the University Health Network Animal Care Committee in accordance with guidelines and regulations set by the Canadian Council on Animal Care.

Note that full information on the approval of the study protocol must also be provided in the manuscript.

Flow Cytometry

Plots

Confirm that:

☒ The axis labels state the marker and fluorochrome used (e.g. CD4-FITC).

☒ The axis scales are clearly visible. Include numbers along axes only for bottom left plot of group (a 'group' is an analysis of identical markers).

☒ All plots are contour plots with outliers or pseudocolor plots.

☒ A numerical value for number of cells or percentage (with statistics) is provided.

Methodology

Sample preparation	Cells were harvested by trypsin treatment and centrifuged at 500 x g for 5 minutes. The pellet was resuspended in ice-cold PBS and centrifuged again. The pellet was then resuspended at a concentration of 4 x 106 cells/ml in the sorting buffer, which is PBS containing 100 Kunitz DNase I/ml, 10 ug/ml propidium iodide (Sigma) and 2% FBS. The sorting solution was also supplemented with either 10 uM forskolin, 100 uM 5,6-Dichlorobenzimidazole riboside (DRB, Sigma), 10 uM forskolin (Sigma) and 100 uM DRB or DMSO, as a control. The cells were then sent through a 40 um filter to remove large clumps and loaded into either a FACScan Flow Cytometer (BD Bioscience) for cell analysis or a FACSVantage SE cell sorter (BD Bioscience) for cell sorting. The cells with positive propidium iodide staining (i.e., dead cells) were first eliminated from the analysis or sorting pool. For cell sorting, the desired population, either the most or least bright EGFP-positive cells, according to the purpose of the experiments (see Results), was sorted Into either 15 ml conical tubes or 96-well plates, which both contained complete DMEM culture media.
Instrument	BD LSR Fortessa: 4 laser (488/640/405/561); 16 parameter analyzer with HTS
Software	FACScan Flow Cytometer (BD Bioscience)
Cell population abundance	More than 100,000 cells were measured for assessing fluorescence intensity for each sample.
Gating strategy	Gating strategy was determined based on the negative cell population.

☒ Tick this box to confirm that a figure exemplifying the gating strategy is provided in the Supplementary Information.
